# Human Enterovirus Nonstructural Protein 2C^ATPase^ Functions as Both an RNA Helicase and ATP-Independent RNA Chaperone

**DOI:** 10.1371/journal.ppat.1005067

**Published:** 2015-07-28

**Authors:** Hongjie Xia, Peipei Wang, Guang-Chuan Wang, Jie Yang, Xianlin Sun, Wenzhe Wu, Yang Qiu, Ting Shu, Xiaolu Zhao, Lei Yin, Cheng-Feng Qin, Yuanyang Hu, Xi Zhou

**Affiliations:** 1 State Key Laboratory of Virology, College of Life Sciences, Wuhan University, Wuhan, Hubei, China; 2 State Key Laboratory of Pathogen and Biosecurity, Beijing Institute of Microbiology and Epidemiology, Beijing, China; University of California, Irvine, UNITED STATES

## Abstract

RNA helicases and chaperones are the two major classes of RNA remodeling proteins, which function to remodel RNA structures and/or RNA-protein interactions, and are required for all aspects of RNA metabolism. Although some virus-encoded RNA helicases/chaperones have been predicted or identified, their RNA remodeling activities *in vitro* and functions in the viral life cycle remain largely elusive. Enteroviruses are a large group of positive-stranded RNA viruses in the *Picornaviridae* family, which includes numerous important human pathogens. Herein, we report that the nonstructural protein 2C^ATPase^ of enterovirus 71 (EV71), which is the major causative pathogen of hand-foot-and-mouth disease and has been regarded as the most important neurotropic enterovirus after poliovirus eradication, functions not only as an RNA helicase that 3′-to-5′ unwinds RNA helices in an adenosine triphosphate (ATP)-dependent manner, but also as an RNA chaperone that destabilizes helices bidirectionally and facilitates strand annealing and complex RNA structure formation independently of ATP. We also determined that the helicase activity is based on the EV71 2C^ATPase^ middle domain, whereas the C-terminus is indispensable for its RNA chaperoning activity. By promoting RNA template recycling, 2C^ATPase^ facilitated EV71 RNA synthesis *in vitro*; when 2C^ATPase^ helicase activity was impaired, EV71 RNA replication and virion production were mostly abolished in cells, indicating that 2C^ATPase^-mediated RNA remodeling plays a critical role in the enteroviral life cycle. Furthermore, the RNA helicase and chaperoning activities of 2C^ATPase^ are also conserved in coxsackie A virus 16 (CAV16), another important enterovirus. Altogether, our findings are the first to demonstrate the RNA helicase and chaperoning activities associated with enterovirus 2C^ATPase^, and our study provides both *in vitro* and cellular evidence for their potential roles during viral RNA replication. These findings increase our understanding of enteroviruses and the two types of RNA remodeling activities.

## Author Summary

Enteroviruses contain a large number of closely related human pathogens, including poliovirus, EV71, and coxsackie viruses, and cause ~3 billion infections annually. Among the nonstructural proteins of enteroviruses or picornaviruses, protein 2C^ATPase^ is the most conserved and complex but the least understood. On the basis of sequence analyses, this protein has been predicted as a putative superfamily 3 (SF3) helicase that supposedly plays a pivotal role in enteroviral RNA replication. However, attempts to determine the helicase activity associated with 2C^ATPase^ have been unsuccessful. We found that eukaryotically expressed EV71 or CAV16 2C^ATPase^ does possess an ATP-dependent RNA helicase activity that 3′→5′ unwinds RNA helices like other SF3 helicases; surprisingly, it also functions as an RNA chaperone that remodels RNA structures in an ATP-independent manner. Moreover, we determined the domain requirements for these two RNA remodeling activities associated with 2C^ATPase^ and provide both *in vitro* and cellular evidence of their potential roles during viral RNA replication. Additionally, our study provides the first evidence that RNA helicase and chaperoning activities can be integrated within one protein, thereby introducing an extended view of RNA remodeling proteins.

## Introduction

For both cells and viruses, the functionality of RNA molecules usually requires correct folding into proper tertiary structures and the association with correct RNA-binding proteins [1,2]. To make the scenario more complex, RNA molecules frequently function via transient base pairing with target RNAs and/or dynamic association/dissociation with distinct RNA-binding proteins [3]. However, correct folding of RNAs is quite challenging and time-consuming, because RNAs can easily become trapped in thermodynamically stable intermediates that are misfolded and inactive. In response, cells and viruses encode a variety of “helper” or RNA remodeling proteins, such as RNA helicases and RNA chaperones, that can destabilize RNA-RNA or RNA-DNA base pairing, thereby lowing the thermodynamic barrier between RNA conformations and promoting the proper formation of RNA tertiary structures [4–6]. It is believed that RNA remodeling proteins play pivotal roles in nearly all processes involving RNA molecules.

RNA helicases are highly similar to DNA helicases, contain ATPase activity, and utilize the energy of ATP binding and/or hydrolysis to translocate along and unwind RNA duplexes. They are thought to participate in most ATP-dependent remodeling of structured RNAs and are classified into six superfamilies (SFs), designated SF1 to SF6, on the basis of conserved helicase motifs [7]. On the other hand, RNA chaperones are a heterogeneous group of proteins that share no consensus sequences or motifs but are able to destabilize RNA duplexes and assist in the formation of more globally stable RNA structures. The major difference between RNA chaperones and helicases is that RNA chaperones do not require ATP binding or hydrolysis for their remodeling activities [8]. (Of note, the definition of RNA chaperones is sometimes blurred, as some publications have defined helicases as a subgroup of ATP-dependent RNA chaperones [2]. Herein, like in most publications, we define RNA helicases and chaperones as two separate classes of the RNA remodelers for the sake of clarity.)

For viruses, particularly RNA viruses, their viral RNAs (vRNAs) contain multiple *cis*-acting elements that play pivotal roles in vRNA replication and translation. These highly structured RNA elements require RNA helicases or chaperones to aid in their proper folding and re-folding. Moreover, during vRNA replication, replicative intermediate double-stranded RNA (dsRNA) must be unwound by virus-encoded or cellular RNA helicases; therefore, vRNA templates can be efficiently re-utilized to synthesize more progeny vRNA strands. Thus far, a variety of RNA viruses, such as flavivirus NS3 [9], alphavirus NSP2 [10], coronavirus nsp13 [11], and alphatetravirus Hel [12], have been reported to encode their own RNA helicases, and the majority of them belong to helicase SF1 and SF2 [7].

**Fig 1:**
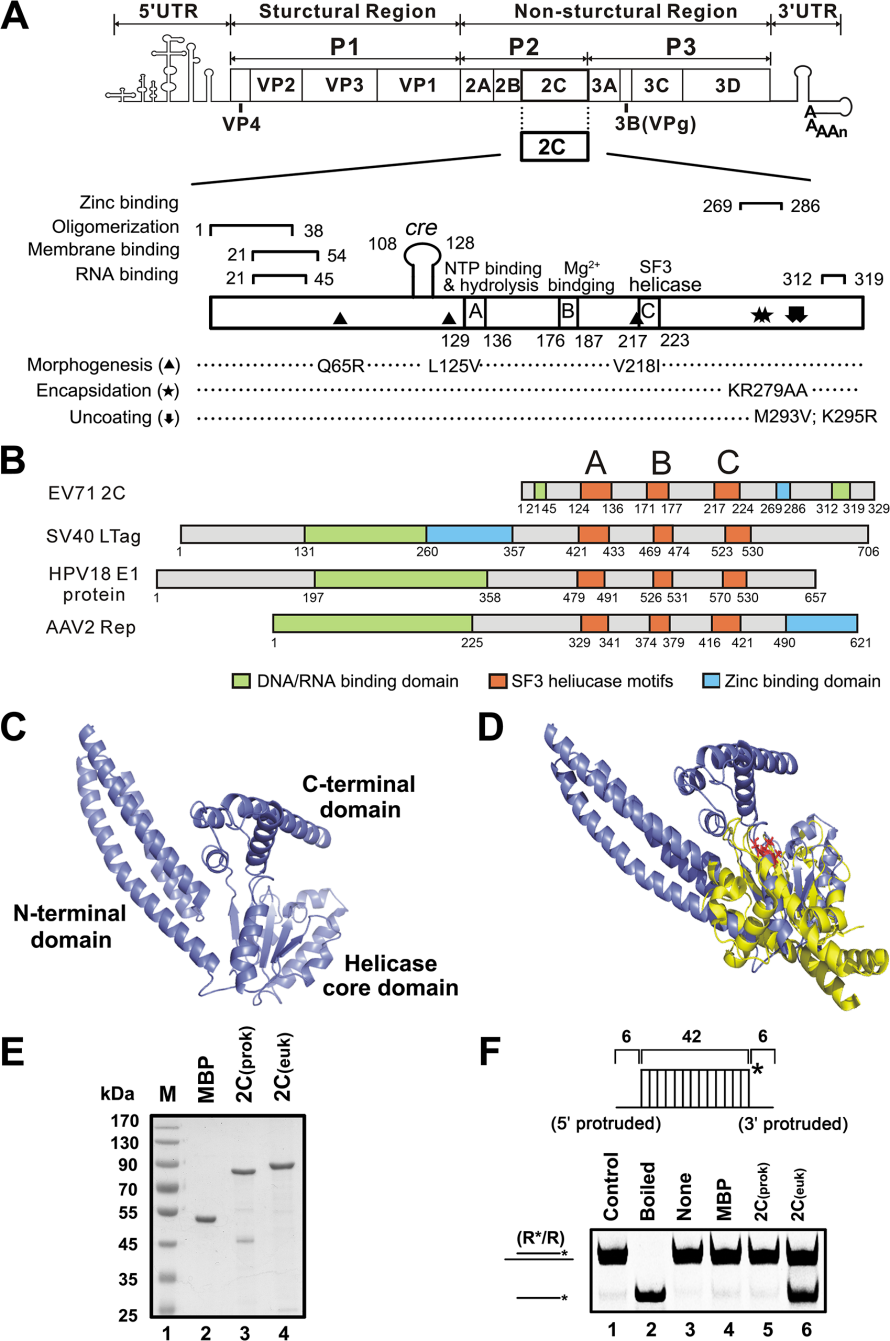
EV71 2C^ATPase^ is similar to other viral SF3 helicases in both motifs and structures. (A) Schematic representation of enterovirus genome and the functional motifs in 2C^ATPase^, including Zinc binding, oligomerization, membrane binding, and RNA binding motifs. Amino acid positions of each motif are numbered. (B) Domain alignments of EV71 2C^ATPase^ and SF3 viral helicases, including SV40 LTag, HPV18 E1, and AAV2 Rep40. SF3 helicase motifs A, B, and C are highlighted in orange. (C) The structure of EV71 2C^ATPase^ was predicted using the HMMSTR/Rosetta server. The N-terminal domain (NTD), the middle helicase core (HC) domain, and the C-terminal domain (CTD) are linked by flexible loops as indicated. (D) Structural alignment of EV71 2C^ATPase^ (slate) and AAV2 Rep40 (yellow). The ATP/ADP binding site is highlighted in red. (E) MBP-fusion EV71 2C^ATPase^ was expressed using a prokaryotic (*E*. *coli*) or eukaryotic (baculovirus) system. The purified recombinant proteins were subjected to 10% SDS-PAGE followed by Coomassie brilliant blue R250 staining. (F) RNA/RNA hybrid helix (R*/R) substrate (0.1 pmol) as illustrated in the upper panel was reacted with 20 pmol prokaryotically (lane 5) or eukaryotically expressed MBP-2C^ATPase^ (lane 6). The helix unwinding activity was detected via gel electrophoresis and scanning on a Typhoon 9200 imager. Native (lane 1) or boiled (lane 2) reaction mixture was used as negative or positive control. Asterisks indicate the HEX-labeled strands.

Enteroviruses are a large group of positive-stranded RNA viruses in the *Picornaviridae* family that contain numerous important human pathogens, including poliovirus, enterovirus 71 (EV71), coxsackie viruses, and echoviruses. They cause approximately 3 billion human infections annually and are responsible for a wide spectrum of diseases, ranging from relatively mild conditions, such as common cold, upper respiratory illness, and hand-foot-and-mouth disease (HFMD), to severe conditions such as aseptic meningitis, encephalitis, myocarditis, neonatal sepsis-like disease, and poliomyelitis [13]. So far, although vaccination has almost eradicated poliomyelitis worldwide [14], no effective vaccine or antiviral therapy is available for many other important human enteroviruses, particularly neurotropic EV71, which is the major causative pathogen for HFMD and a serious lethal threat to children. Enterovirus encodes a single polyprotein that is proteolytically cleaved into separate structural and nonstructural proteins. Among them, the multifunctional protein 2C^ATPase^ is the most conserved and complex but the least understood (Fig 1A). Enterovirus 2C^ATPase^ has been reported to participate in diverse processes, including RNA binding [15,16], RNA replication [17–19], membrane anchoring and rearrangement [20,21], autophagy inhibition [22], encapsidation and viral morphogenesis [23,24], and suppression of nuclear factor kappa B activation [25]. 2C^ATPase^ has long been predicted as a putative SF3 helicase on the basis of its AAA+ ATPase activity and conserved SF3 signature motifs (Fig 1B) [26,27]. Similar to other well-defined SF3 helicases, such as Simian virus 40 (SV40) large T antigen (LTag), human papillomavirus (HPV) E1, and adeno-associated virus (AAV) Rep, enterovirus 2C^ATPase^ has been reported to form homo-oligomeric ring-like structures *in vitro*, which is required for its ATPase activity [28]. Moreover, this protein appears to associate with vRNA in the vRNA replication complexes in infected cells [29], implying that 2C^ATPase^ directly participates in enteroviral RNA replication. However, previous attempts to determine the helicase activity associated with 2C^ATPase^ or other enteroviral proteins have been unsuccessful [27]. Thus, whether enteroviral or picornaviral RNA replication requires the involvement of any virus-encoded RNA remodeling activity is not known, and this gap hinders our understanding of this large group of medically important pathogenic viruses.

Interestingly, our previous study of the 2C^ATPase^ protein from *Ectropis obliqua* picorna-like virus (EoV; genus *Iflavirus*, family *Iflaviridae*, order *Picornavirales*) revealed that EoV 2C^ATPase^ possesses RNA remodeling activity that is more like the activity of an RNA chaperone since EoV 2C^ATPase^ can destabilize RNA duplexes and accelerate strand annealing in an ATP-independent manner [30]. This finding obtained from an insect picorna-like virus prompted us to determine whether enterovirus 2C^ATPase^ could be an RNA remodeler. Herein, we report that although bacterially expressed EV71 2C^ATPase^ did not exhibit any RNA remodeling activity, eukaryotically expressed EV71 2C^ATPase^ functions not only as an RNA helicase that unwinds RNA helices from 3′ to 5′ in an ATP-dependent manner but also as an RNA chaperone that destabilizes helices from either direction and facilitates strand annealing and complex RNA structure formation independently of ATP. Moreover, we determined the domain requirements for these two RNA remodeling activities associated with 2C^ATPase^ and showed that protein 2C^ATPase^ facilitates the EV71 RNA-dependent RNA polymerase (RdRP)-mediated synthesis of vRNA from vRNA template *in vitro*. When the helicase activity of 2C^ATPase^ was disrupted by point mutation, the RNA replication and virus production of EV71 were mostly abolished in cells. These data indicate that 2C^ATPase^-mediated RNA remodeling plays a critical role in the enteroviral life cycle, particularly in enteroviral replication. In addition, our data show that the RNA helicase and chaperoning activities are also conserved in CAV16 2C^ATPase^.

## Results

### Enterovirus 2C^ATPase^ is similar to other viral SF3 helicases in both conserved motifs and structures

To evaluate the potential helicase or RNA remodeling activity of an enterovirus 2C^ATPase^, we first compared the sequences and consensus motifs of EV71 2C^ATPase^ and three other viral SF3 helicases: SV40 LTag, HPV18 E1, and AAV2 Rep40. Although EV71 2C^ATPase^ is smaller, this protein contains a core helicase region and the conserved SF3 signature A, B, and C motifs, similar to the three other SF3 helicases (Fig 1B).

To further analyze the potential function of 2C^ATPase^, we modeled its three-dimensional (3D) structure using ROBETTA [31]. The computation-generated structural modeling of 2C^ATPase^ indicated that this protein may be comprised of three structurally independent domains: the N-terminal domain (NTD) contains 113 amino acids (a.a.) and is predicted as a helix bundle formed by three α helices; the middle helicase core (HC) domain includes a.a. 114–250 of 2C^ATPase^, and is predicted as a central five-stranded β-sheet flanked by α helices on both sides; and the C-terminal domain (CTD) includes a.a. 251–329 and contains several α helices. The three domains are linked by flexible loops (Fig 1C). The crystallographic structures of AAV2 Rep40 and Rep40-ADP complex have been previously resolved [32,33]. Our structural alignment of EV71 2C^ATPase^ and AAV2 Rep40 shows that the predicted HC domain of 2C^ATPase^ is structurally similar with the counterpart region of Rep40. Moreover, similarly with Rep40, 2C^ATPase^ is also predicted to contain a P-loop connecting with the central β-sheet, and the predicted ATP binding site of 2C^ATPase^ is also exposed to the exterior and perfectly overlapped with the structurally determined ATP/ADP binding site of Rep40 in the structural alignment of these two proteins (Fig 1D). Altogether, our sequence and structural analyses indicate that enterovirus 2C^ATPase^ is similar to other viral SF3 helicases in both conserved motifs and structures, implying that this protein should contain some RNA remodeling activity.

### EV71 2C^ATPase^ can unwind both RNA and DNA helices

To assess the potential RNA remodeling activity of enterovirus 2C^ATPase^, we first expressed EV71 2C^ATPase^ as an MBP-fusion protein in both prokaryotic (*E*. *coli*) and eukaryotic (baculovirus) systems (Fig 1E). Interestingly, the eukaryotically expressed MBP-2C^ATPase^ had an apparently higher molecular mass than the prokaryotically expressed one (Fig 1E, lanes 4 vs. 3). The identity and purity of the eukaryotically expressed MBP-2C^ATPase^ were further confirmed via SDS-PAGE separation followed by silver staining (S1 Fig) or in-gel digestion and high-performance Liquid chromatography-tandem mass spectrometry (LC-MS/MS) analysis (S1 Table and S1 Text). In total, four proteins (MBP-2C^ATPase^, α-tubulin, actin and cytochrome P450) were identified with FDR value of 0.97%. MBP-2C^ATPase^ turned out to be the highest scoring protein (score: 11200) identified with high protein coverage of 81.6%. The mass spectrometry results showed that the majority (97.8%) of all the spectra obtained in the purified protein sample were originated from MBP-2C^ATPase^. The quantitation information by Mascot Exponentially Modified Protein Abundance Index (emPAI) further confirmed the purity of our target protein: the emPAI value of MBP-2C^ATPase^ is 126.76, in comparison to the other three identified proteins including α-tubulin, actin and cytochrome P450 from *Spodoptera frugiperda* Sf9 cell with very low emPAI values of 0.65, 0.11, 0.07, respectively. Furthermore, to examine the oligomerization state of MBP-2C^ATPase^, the purified eukaryotically expressed MBP-2C^ATPase^ was subjected to the size exclusion chromatography analysis using a Superdex 200 column. The major peak comprised most protein that was eluted as a molecular mass of ~600 kDa (S2A Fig), and the eluted protein was ~85 kDa in size as determined by 10% SDS-PAGE and silver staining (S2B Fig), indicating that the eukaryotically expressed MBP-2C^ATPase^ is oligomerized, consistent with previous studies of poliovirus 2C^ATPase^ [28].

**Fig 2:**
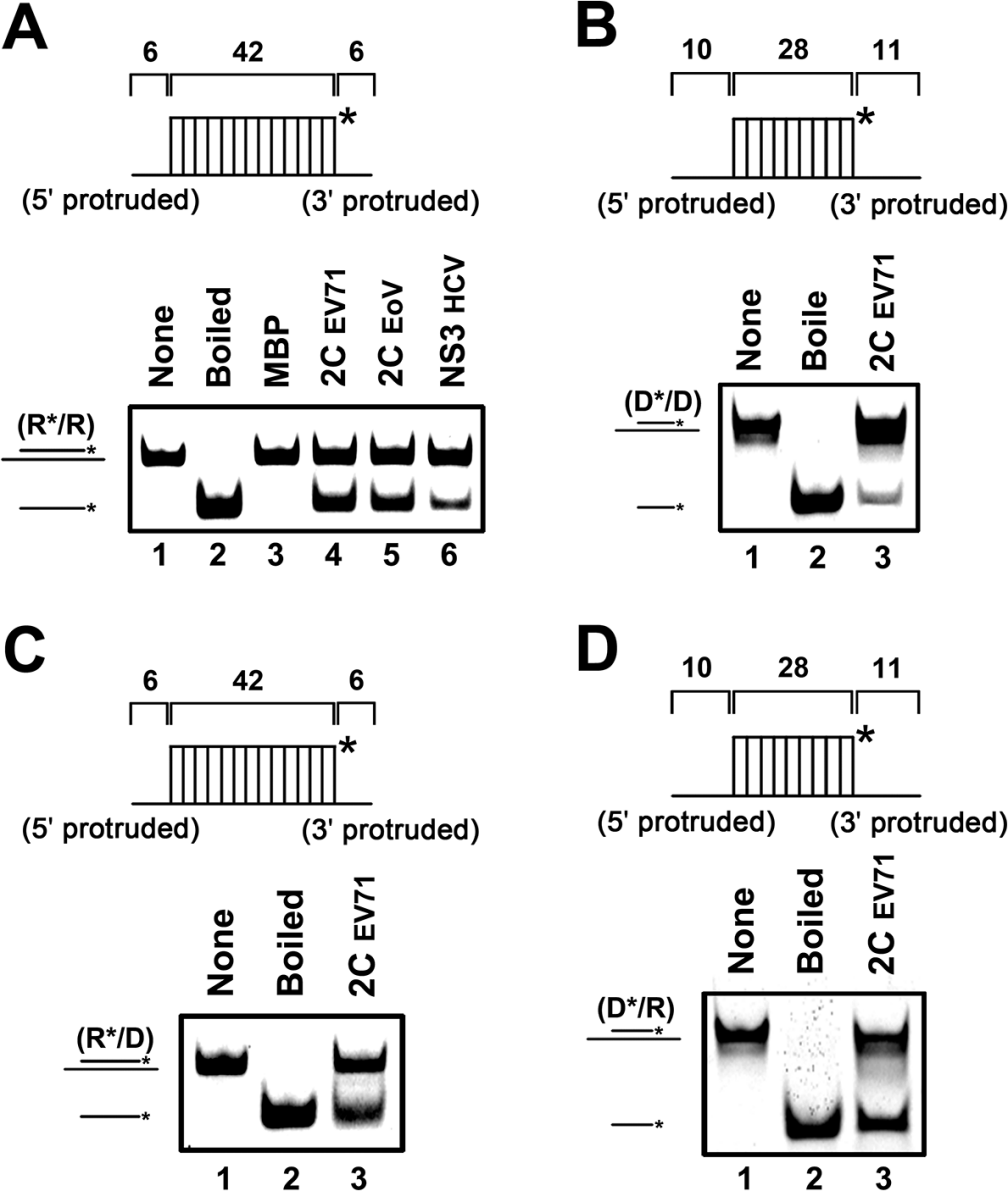
EV71 2C^ATPase^ unwinds both RNA and DNA helices. **(A)** The standard RNA/RNA hybrid helix (R*/R) substrate (0.1 pmol) as illustrated in the upper panel was reacted with each indicated protein (20 pmol). The helix unwinding activity was detected via gel electrophoresis and scanning on a Typhoon 9200 imager. Lane 1, reaction mixture without 2C^ATPase^; lane 2, boiled reaction mixture without 2C^ATPase^; lane 3, complete reaction mixture with MBP alone; lane 4, complete reaction mixture with MBP-fusion EV71 2C^ATPase^; lane 5, complete reaction mixture with MBP-fusion EoV 2C^ATPase^; lane 6, complete reaction mixture with MBP-fusion HCV NS3. **(B)** to **(D)** As illustrated in each upper panel, 0.1 pmol DNA/DNA (D*/D) (B), RNA/DNA (R*/D) (C), or DNA/RNA (D*/R) (D) substrate was incubated in the presence (lane 3) or absence (lane 1) of MBP-fusion EV71 2C^ATPase^. Asterisks indicate the HEX-labeled strands.

To evaluate the helix unwinding activity of EV71 2C^ATPase^, a short hexachloro fluorescein (HEX)-labeled RNA and a long non-labeled RNA were annealed to generate a standard RNA helix substrate with both 5′ and 3′ single-stranded protrusions (Fig 1F, upper panel and S2 Table). The helix unwinding assay was conducted by incubating the RNA helix substrate with purified MBP-2C^ATPase^, followed by gel electrophoresis. Interestingly, our data showed that, although bacterially expressed MBP-2C^ATPase^ did not unwind the RNA helix in the presence of MgCl_2_ and ATP (Fig 1F, lane 5), consistent with observations reported previously by another group [27], eukaryotically expressed MBP-2C^ATPase^ efficiently unwound the RNA helix substrate under the same conditions (Fig 1F and Fig 2A). Of note, EoV 2C^ATPase^ and Hepatitis C virus (HCV) NS3 [30,34] were used as positive controls in this assay (Fig 2A). Moreover, EV71 2C^ATPase^ could unwind the RNA duplex with 42 matched base pairs but not the duplex with 49 matched base pairs (S3 Fig), as the latter substrate might be too long to be unwound. Altogether, these results indicate that EV71 2C^ATPase^ can unwind an RNA helix when the protein is eukaryotically expressed.

Because some RNA helicases or chaperones also show unwinding activity with DNA helices or RNA-DNA hybrids, we evaluated this possibility for EV71 2C^ATPase^. To this end, we constructed three different helix substrates that were DNA helix (Fig 2B) and RNA-DNA hybrids with longer DNA (Fig 2C) or RNA strand (Fig 2D). Each helix substrate was reacted with MBP-2C^ATPase^ under the same conditions as illustrated in Fig 1F. Our data showed that EV71 2C^ATPase^ could unwind all of the tested types of helix substrates (Fig 2), although it unwound the DNA helix less efficiently than it unwound other helices (Fig 1F). Thus, we conclude that EV71 2C^ATPase^ possesses a nucleic acid helix unwinding activity for both RNA and DNA duplexes.

### EV71 2C^ATPase^ directs both 5′→3′ and 3′→5′ unwinding of RNA helices

**Fig 3:**
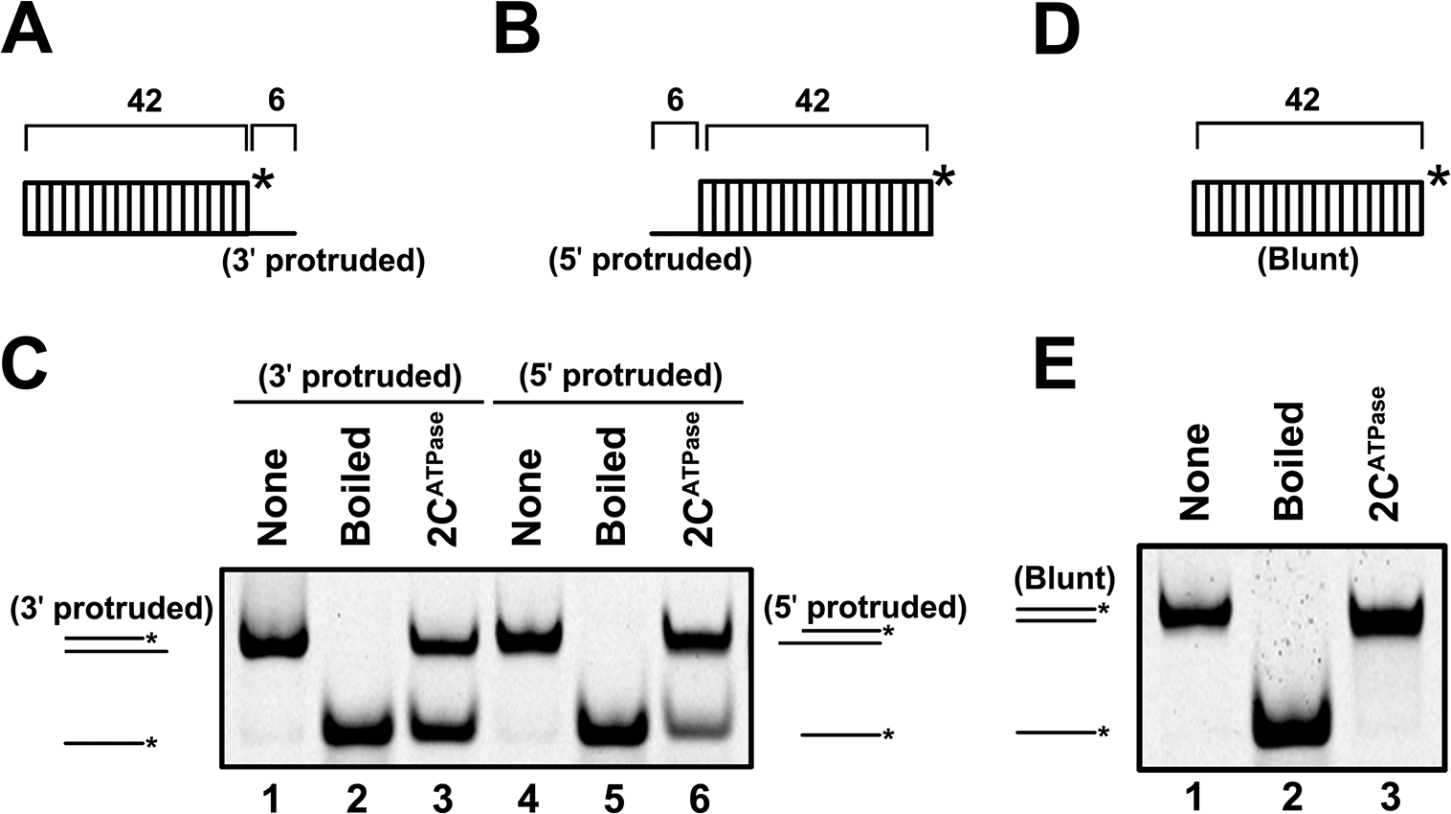
2C^ATPase^ unwinds RNA helices in a bidirectional manner. **(A)** and **(B)** Schematic illustration of the RNA helix substrate with the 3′ single-stranded protrusion (6 bases) (A) or 5′ single-stranded protrusion (6 bases) (B). Asterisks indicate the HEX-labeled strands. **(C)** MBP-2C^ATPase^ (20 pmol) was reacted with 3′-protruded (lane 3) or 5′-protruded (lane 6) RNA helix substrate (0.1 pmol). Native (lanes 1 and 4) or boiled reaction mixture (lanes 2 and 5) was used as a negative or positive (lane 2 and 5) control, respectively. **(D)** Schematic illustration of the blunt-ended RNA helix substrate. **(E)** The blunt-ended RNA helix (0.1 pmol) was reacted with MBP-2C^ATPase^ (20 pmol).

For helicases, the directionality of helix unwinding is a fundamental characteristic [35]. To assess the unwinding directionality of EV71 2C^ATPase^, we constructed three different RNA helix substrates containing a 3′ single-stranded protrusion, a 5′ single-stranded protrusion, and blunt ends, respectively (Fig 3A, 3B and 3D). Each substrate was then incubated with 2C^ATPase^ for the helix unwinding assay. Our results showed that EV71 2C^ATPase^ could unwind either the 3′- or 5′-protruded RNA helix (Fig 3C), and its efficiency to unwind the 3′-protruded helix was apparently higher than its efficiency to unwind the 5′-protruded one (Fig 3C, lanes 3 vs. 6). Moreover, 2C^ATPase^ could not unwind the blunt-ended helix (Fig 3E). All these experiments were independently repeated several times. Based on these results, we conclude that EV71 2C^ATPase^ possesses a bidirectional unwinding activity to RNA helices, while the 3′→5′ unwinding is more preferred.

### The helix unwinding activity of 2C^ATPase^ can be promoted by the presence of ATP

For helicases, one important property is that their helix-unwinding activities require the presence of ATP, and ATP promotes helicase activities in a dose-dependent manner [36]. Our data showed that EV71 2C^ATPase^ hydrolyzed ATP or GTP, while UTP or CTP was not efficiently hydrolyzed by this protein. In addition, ssRNA had little effect on the NTPase activity of 2C^ATPase^ (S4A Fig).

**Fig 4:**
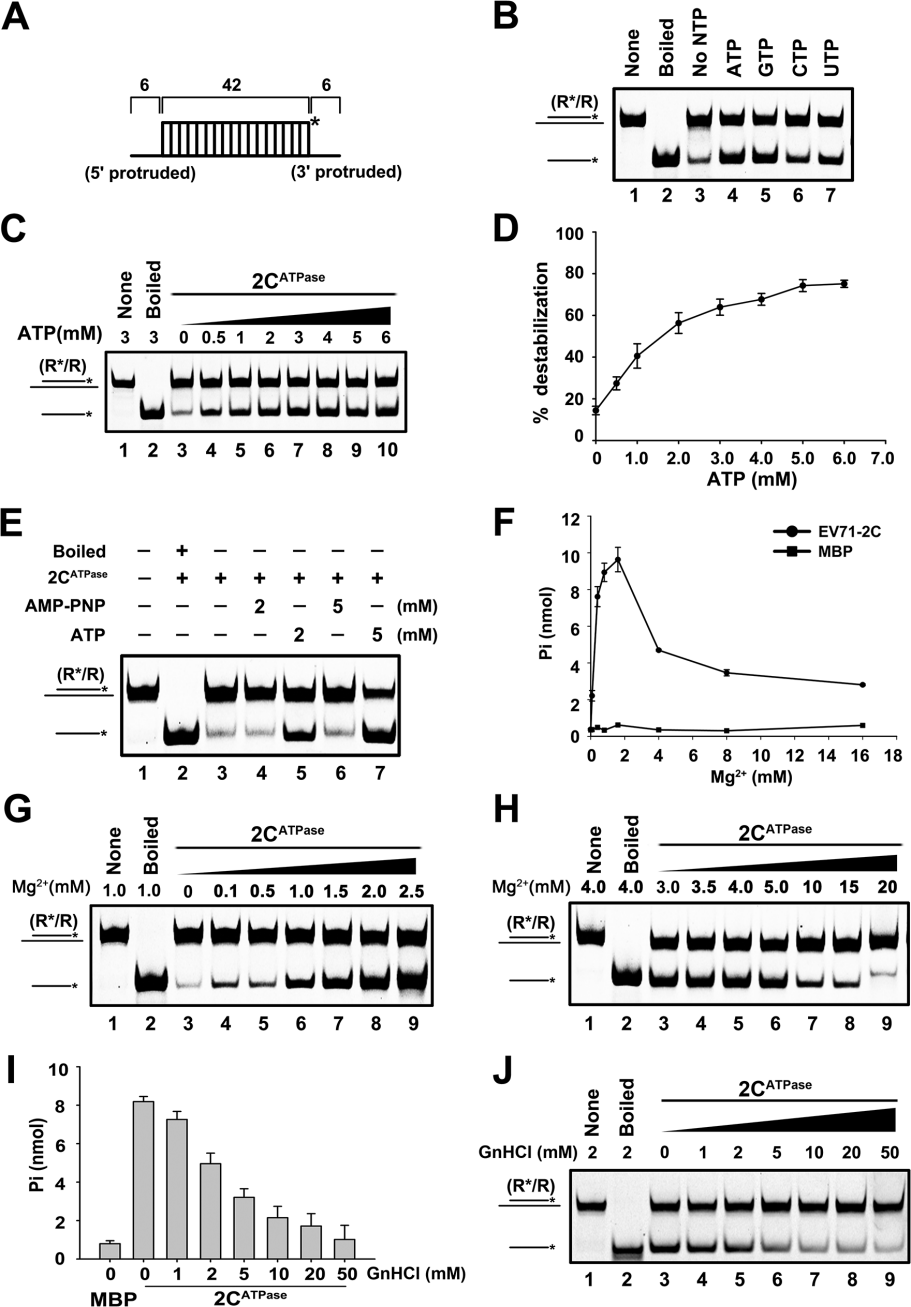
EV71 2C^ATPase^ possesses both ATP-dependent and ATP-independent helix unwinding activities. **(A)** Schematic illustration of the standard RNA helix substrate that contains both 3′- and 5′-protrusions. **(B)** and **(C)** The standard RNA helix (0.1 pmol) was reacted with MBP-2C^ATPase^ in the absence or presence of the indicated NTPs (5 mM) (B) or in the presence of increasing concentrations (0–6 mM) of ATP as indicated (C). **(D)** The unwinding activities under different ATP concentrations were plotted as the percentage of the released RNA from the total RNA helix substrate (Y-axis) at each ATP concentration (X-axis). Error bars represent standard deviation (SD) values from three separate experiments. **(E)** Unwinding assays of 2C^ATPase^ using the standard helix substrate were performed in the absence or presence of ATP or ATP analog (AMP-PNP) as indicated. **(F)** The ATPase activity of MBP-2C^ATPase^ was measured as nanomoles of released inorganic phosphate at the indicated Mg^2+^ concentrations. Error bars represent SD values from three separate experiments. **(G)** and **(H)** Unwinding assays of 2C^ATPase^ were performed in the presence of 0–2.5 mM (G) or 3–20 mM (H) Mg^2+^ as indicated. **(I)** The ATPase activity of MBP-2C^ATPase^ was measured as nanomoles of released inorganic phosphate at the indicated GnHCl concentrations. Error bars represent SD values from three separate experiments. **(J)** Unwinding assays of 2C^ATPase^ were performed in the presence of 0–50 mM GnHCl as indicated.

To determine the effect of NTPs on 2C^ATPase^, we conducted a standard unwinding assay in the presence or absence of individual NTPs. Our results showed that the presence of ATP or GTP dramatically promoted helix unwinding (Fig 4B). To determine whether the promoting effect of ATP on helix unwinding by 2C^ATPase^ is dose-dependent like other helicases, we assessed the unwinding activity of MBP-2C^ATPase^ in the presence of increasing ATP concentrations from 0.5 to 6 mM. Our data showed that increasing ATP concentrations apparently promoted helix unwinding, and the promoting effect reached a steady stage at 5 mM ATP (Fig 4C and 4D). These results indicate that the helix unwinding activity of EV71 2C^ATPase^ needs the participation of ATP or other NTPs.

### EV71 2C^ATPase^ possesses both ATP-dependent and ATP-independent helix unwinding activities

Although we determined that ATP is required by EV71 2C^ATPase^ to reach its optimal unwinding activity, very interestingly, this protein was still able to unwind a portion of RNA helix in the absence of ATP (Fig 4B, lane 3; Fig 4C, lane 3; and Fig 4D). To confirm this phenomenon and exclude the potential interference of trace amounts of ATP in the reactions, we added AMP-PNP, a non-hydrolyzable ATP analog that blocks ATPase activity, to the ATP-free unwinding reaction. Our data showed that although the presence of 5 mM AMP-PNP was able to abolish the unwinding activity of HCV NS3, a classic RNA helicase (S4B Fig), the presence of 2 or 5 mM AMP-PNP was unable to completely block 2C^ATPase^ to unwind a portion of the RNA helix (Fig 4E, lanes 4 and 6).

To further exploit this issue, we sought to determine the necessity of Mg^2+^ on the ATPase and helix unwinding activities of EV71 2C^ATPase^, as these two closely coupled activities of a helicase is normally Mg^2+^-dependent [37]. Our results showed that the ATPase activity of EV71 2C^ATPase^ was strictly Mg^2+^-dependent, as this protein was almost unable to hydrolyze ATP in the absence of Mg^2+^ (Fig 4F). On the other hand, although 2C^ATPase^ requires the presence of Mg^2+^ to reach its optimal unwinding activity, it was still able to unwind a portion of the RNA helix in the absence of Mg^2+^ or any divalent ions (Fig 4G, lane 3; S4D Fig, lane 3), further confirming that 2C^ATPase^ retains partial helix unwinding activity in the absence of its ATPase activity. Moreover, when the Mg^2+^ concentration was lower than 2 or 2.5 mM, increasing Mg^2+^ concentrations gradually promoted both the ATPase and helix unwinding activities of EV71 2C^ATPase^ (Fig 4F and 4G), whereas at higher Mg^2+^ concentrations (>2.5 mM), Mg^2+^ inhibited both activities in a dose-dependent manner (Fig 4F and 4H). Of note, both no protein supplementation and boiled MBP-2C^ATPase^ were used as negative controls (S4C Fig, lanes 1 and 5). In addition, the biochemical properties of EV71 2C^ATPase^, including the requirement of divalent ions (Mn^2+^, Ca^2+^, and Zn^2+^) and pH, were also determined (S4D–S4F Fig). Our data showed that while 2.5 mM Ca^2+^ is dispensable for the helix unwinding activity of EV71 2C^ATPase^, 2.5 mM Mn^2+^ or Zn^2+^ blocked the helix unwinding by 2C^ATPase^ (S4D Fig). When the concentration of Zn^2+^ is higher than 0.5 mM, the presence of Zn^2+^ completely blocked the helix unwinding activity of 2C^ATPase^ (S4E Fig). Moreover, EV71 2C^ATPase^ prefers a neutral pH as an optimal reaction condition, as this protein exhibited the highest helix unwinding activity with pH 7.5 (S4F Fig).

Previous studies reported that the ATPase activity of poliovirus 2C^ATPase^ can be inhibited by guanidine hydrochloride (GnHCl) [26], which is a potent inhibitor of enterovirus VPg-uridylation and vRNA replication [38,39]. Our data showed that GnHCl can also inhibit the ATPase and RNA helix unwinding activities of EV71 2C^ATPase^ in a dose-dependent manner (Fig 4I and 4J). Interestingly, this protein could still unwind a portion of the RNA helix even at high concentrations of GnHCl, consistent with the results of AMP-PNP treatment (Fig 4E).

Altogether, our results show that EV71 2C^ATPase^ contains an ATP-dependent helicase-like activity that requires the presence of Mg^2+^, together with an ATP/Mg^2+^-independent non-helicase activity that can also unwind the RNA helix.

### EV71 2C^ATPase^ contains both RNA helicase and chaperoning activities

RNA remodeling proteins include two distinct classes: RNA helicases and RNA chaperones. Their fundamental characteristics and differences are that the helix unwinding of RNA helicases is ATP dependent and normally has directionality, whereas that of RNA chaperones does not require ATP and is bidirectional [40]. Our previous results showed that in addition to its ATP-dependent helicase-like activity, EV71 2C^ATPase^ also contains an ATP-independent helix unwinding activity (Fig 4); moreover, this protein can unwind RNA helices bidirectionally (Fig 3C). These findings suggested that EV71 2C^ATPase^ contains both RNA helicase and chaperone activities.

**Fig 5:**
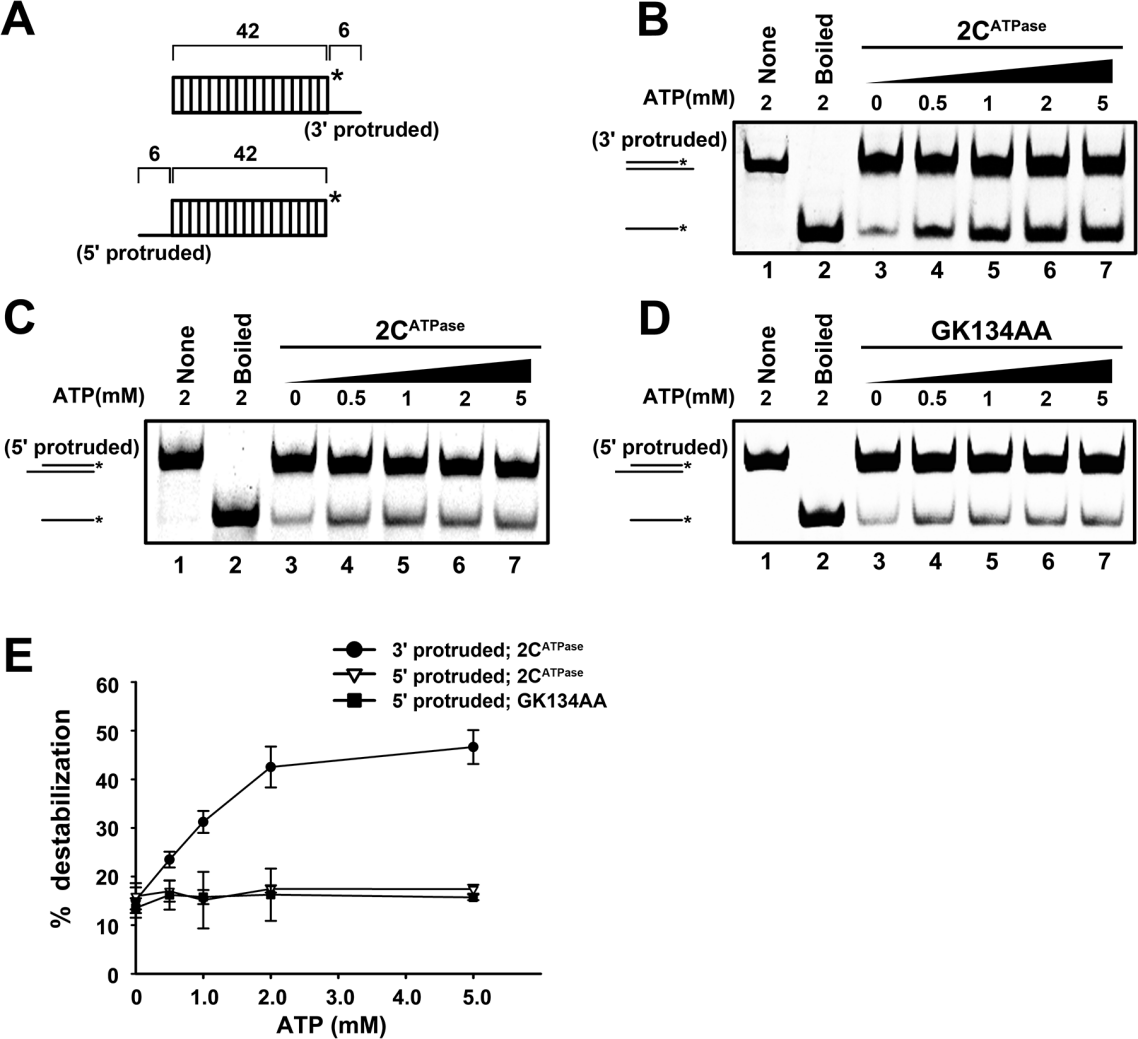
EV71 2C^ATPase^ contains both RNA helicase and chaperone activities. **(A)** Schematic illustration of the RNA helix substrate with the 3′ single-stranded (upper panel) or 5′ single-stranded (lower panel) protrusion. **(B)** The 3′-protruded RNA helix (0.1 pmol) was reacted with MBP-2C^ATPase^ (20 pmol) in the presence of increasing concentrations of ATP as indicated (lanes 3–7). **(C)** and **(D)** The 5′-protruded RNA helix (0.1 pmol) was reacted with MBP-fusion Wt (C) or GK134AA mutant 2C^ATPase^ (D) in the presence of increasing concentrations of ATP as indicated (lanes 3–7). **(E)** For each indicated RNA helix substrate, the unwinding activities of Wt or GK134AA mutant 2C^ATPase^ at different ATP concentrations were plotted as the percentage of the released RNA from the total RNA helix substrate (Y-axis) at each ATP concentration (X-axis). Error bars represent SD values from three separate experiments.

To distinguish these two activities, we designed a set of experiments based on their fundamental difference in helix unwinding directionality and ATP dependency. First, we constructed two RNA helix substrates, each of which contained the same 42-bp double-stranded region as well as a 6-nt 3′ or 5′ single-stranded protrusion (Fig 5A). Then, each helix substrate was reacted with MBP-2C^ATPase^ in the absence or presence of ATP. Our results showed that although the 2C^ATPase^ unwound the 3′-protruded helix in an ATP-dependent manner (Fig 5B and 5E), it unwound the 5′-protruded helix exactly like an RNA chaperone, which was able to unwind the helix in the absence of ATP, and increasing ATP concentrations could not further enhance the helix unwinding (Fig 5C and 5E). Moreover, since the motif A “GKS” is recognized as the NTP binding site and the core ATPase site of SF3 helicases, we generated the ATPase-defective GK134AA mutant of 2C^ATPase^. Our data showed that the GK134AA 2C^ATPase^ mutant was able to unwind the 5′-protruded helix substrate in the absence or presence of ATP at an efficiency similar to that of wild-type 2C^ATPase^, further confirming that the unwinding of the 5′-protruded helix by 2C^ATPase^ is independent of its ATPase activity (Fig 5D and 5E). Furthermore, the unwinding activity of 2C^ATPase^ on the 5′-protruded helix could not be promoted by any individual NTP (S5 Fig). Altogether, our data indicate that the 5′-protruded helix could be unwound via the RNA chaperone activity but not the helicase activity of 2C^ATPase^, whereas the 3′-protruded helix substrate could be unwound via both activities. These findings are consistent with our previous observation that 2C^ATPase^ unwinds the 3′-protruded RNA helix more efficiently than the 5′-protruded one in the presence of ATP (Fig 3C).

We conclude that EV71 2C^ATPase^ possesses an ATP-dependent RNA helicase activity that unwinds RNA helices with 3′**→**5′ directionality, which is consistent with all known SF3 helicases [41], together with an ATP-independent RNA chaperone activity that unwinds RNA helices from either direction.

### EV71 2C^ATPase^ destabilizes structured RNA strands and stimulates annealing

**Fig 6:**
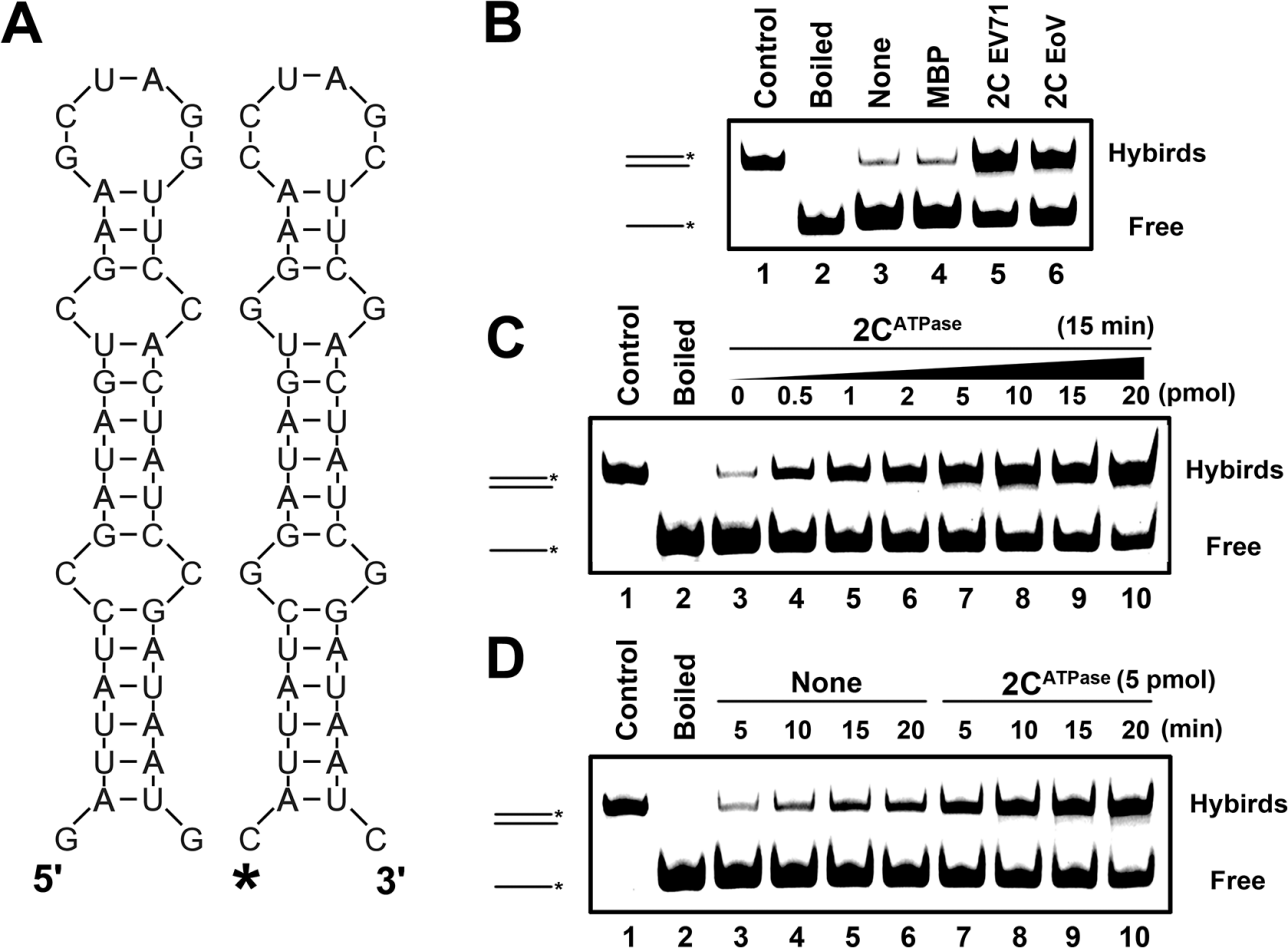
2C^ATPase^ destabilizes structured RNA strands and stimulates annealing. **(A)** Schematic illustration of the stem-loop structures of the two complementary 42-nt RNA substrates. Asterisk indicates the HEX-labeled strand. **(B)** The two strands were 1:1 mixed (0.1 pmol each) and reacted with 5 pmol each indicated protein. **(C)** The two strands were mixed (0.1 pmol each) and reacted with increasing amounts (0–20 pmol) of MBP-fusion EV71 2C^ATPase^ as indicated. **(D)** The hybridization assay as in (B and C) was performed in the absence (lanes 3–6) or presence (lanes 7–10) of 5 pmol MBP-fusion EV71 2C^ATPase^ for different reaction times (5–20 min) as indicated. For (B-D), the mix of two strands was preannealed (lane 1) or boiled (lane 2) as a positive or negative control, respectively. The hybridized and free strands are indicated.

RNA chaperones are generally thought to destabilize misfolded RNAs and assist in the formation of more globally stable RNA structures. After determining that EV71 2C^ATPase^ possesses an RNA chaperone activity, we adapted a canonical assay [42] to further verify and characterize EV71 2C^ATPase^. Two 42-nt complementary RNA strands, each of which formed defined stem-loop structures, were designed and constructed (Fig 6A). Of the two, one strand was 5′ HEX-labeled, and the other was not labeled as indicated. Equal amounts of HEX-labeled and non-labeled strands were incubated in the presence or absence of MBP-2C^ATPase^, and then gel shift assays were conducted to examine the hybridization of the two strands. Of note, all the experiments were ATP-free. Our results showed that although minimal spontaneous hybridization could be observed in the absence of 2C^ATPase^ (Fig 6B, lanes 3–4; Fig 6C, lane 3; and Fig 6D, lanes 3–6), the presence of EV71 2C^ATPase^ dramatically enhanced the two strands' hybridization, as did EoV 2C, which is a well-defined RNA chaperone (Fig 6B, lanes 5–6). Moreover, increasing either the amount of 2C^ATPase^ or its incubation time resulted in an apparent increase in strand annealing (Fig 6C and 6D). Overall, our data show that 2C^ATPase^ can destabilize structured RNA strands and accelerate the formation of more stable hybrids, fitting the characteristic of RNA chaperones.

### EV71 2C^ATPase^ enhances hammerhead ribozyme activity

**Fig 7:**
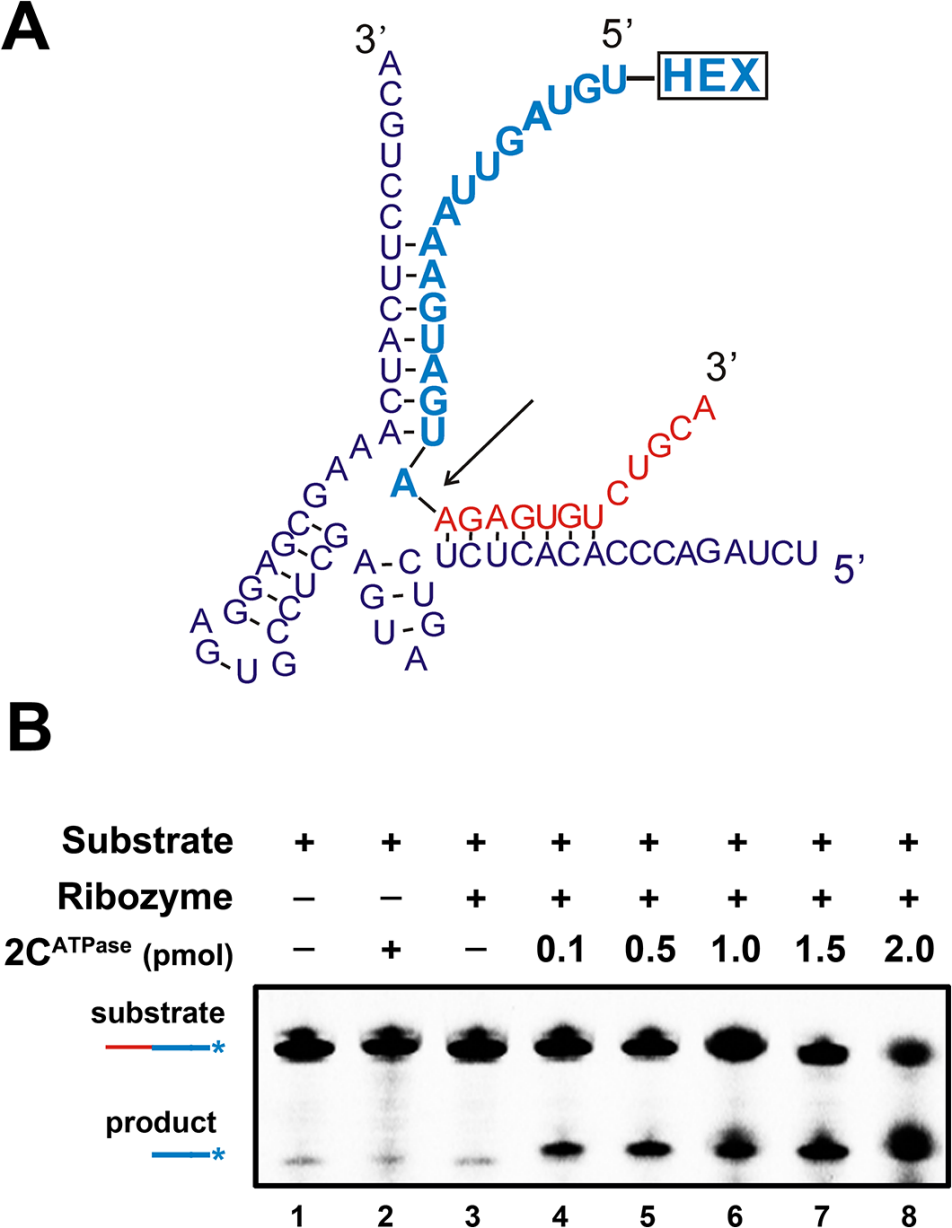
2C^ATPase^ enhances hammerhead ribozyme activity. **(A)** Schematic illustrations of the sequences and secondary structures of hammerhead ribozyme (purple) and its substrate RNA (red and blue). The ribozyme is a 58-nt unlabeled RNA synthesized by T7 RNA polymerase, and the 28-nt RNA substrate was synthesized with its 5′ end being HEX labeled. Under the correct structure of the hammerhead ribozyme, the cleavage of the substrate takes place between the 12^th^ and 13^th^ nucleotides from the 3′ end (indicated by arrow). **(B)** The RNA substrate was incubated with the ribozyme in the absence (lane 3) or presence (lanes 4–8) of increasing amounts (0.1–2 pmol) of MBP-2C^ATPase^ as indicated. The HEX-labeled 16-nt cleavage product (blue) was detected via denaturing gel electrophoresis and scanning. Non-ribozyme supplementation in the absence (lane 1) or presence (lane 2) of 2C^ATPase^ was used as a negative control. The non-cleaved substrate and cleaved product strands are indicated.

The hammerhead ribozyme is a self-catalytic RNA module that mediates reversible *trans*-cleavage at a specific site within an RNA substrate [43]. Since the functionality of a hammerhead ribozyme strictly relies on its correct tertiary structure, hammerhead ribozymes often serve as the models for studying the structure and properties of RNAs. RNA chaperones are probably able to enhance the activity and turnover of ribozymes by promoting ribozyme formation, ribozyme-substrate association, and/or ribozyme dissociation from cleaved substrates. Therefore, a canonical hammerhead ribozyme enhancement assay [44] was used to further verify and characterize the RNA chaperone activity of EV71 2C^ATPase^. In this assay, unlabeled ribozyme RNA was reacted with a 5′ HEX-labeled RNA substrate, and the cleavage was expected to take place 16 nts from the 5′ end (indicated by arrow), resulting in a HEX-labeled 16-nt cleavage product that could be detected by electrophoresis (Fig 7A). Our data showed that the presence of 2C^ATPase^ effectively enhanced ribozyme activity in a dose-dependent manner (Fig 7B), indicating that the RNA chaperone activity of EV71 2C^ATPase^ can assist in the proper formation and function of relatively complex RNA structures.

### The CTD of 2C^ATPase^ is required for its RNA chaperoning activity

The structure of enterovirus 2C^ATPase^ was previously modeled to contain three domains: the N-terminal domain (NTD), helicase core, and CTD (Fig 1C). To assess the role of specific domains on the different activities of 2C^ATPase^, a series of truncations of EV71 2C^ATPase^ were generated and eukaryotically expressed. Moreover, since the motif A “GKS” and motif C “STN” are conserved in SF3 helicases, the GK134AA and ΔSTN mutants of 2C^ATPase^ were also generated. The predicted structures of these 2C^ATPase^ fragments and mutagenesis sites are illustrated in S6A–S6C Fig

**Fig 8:**
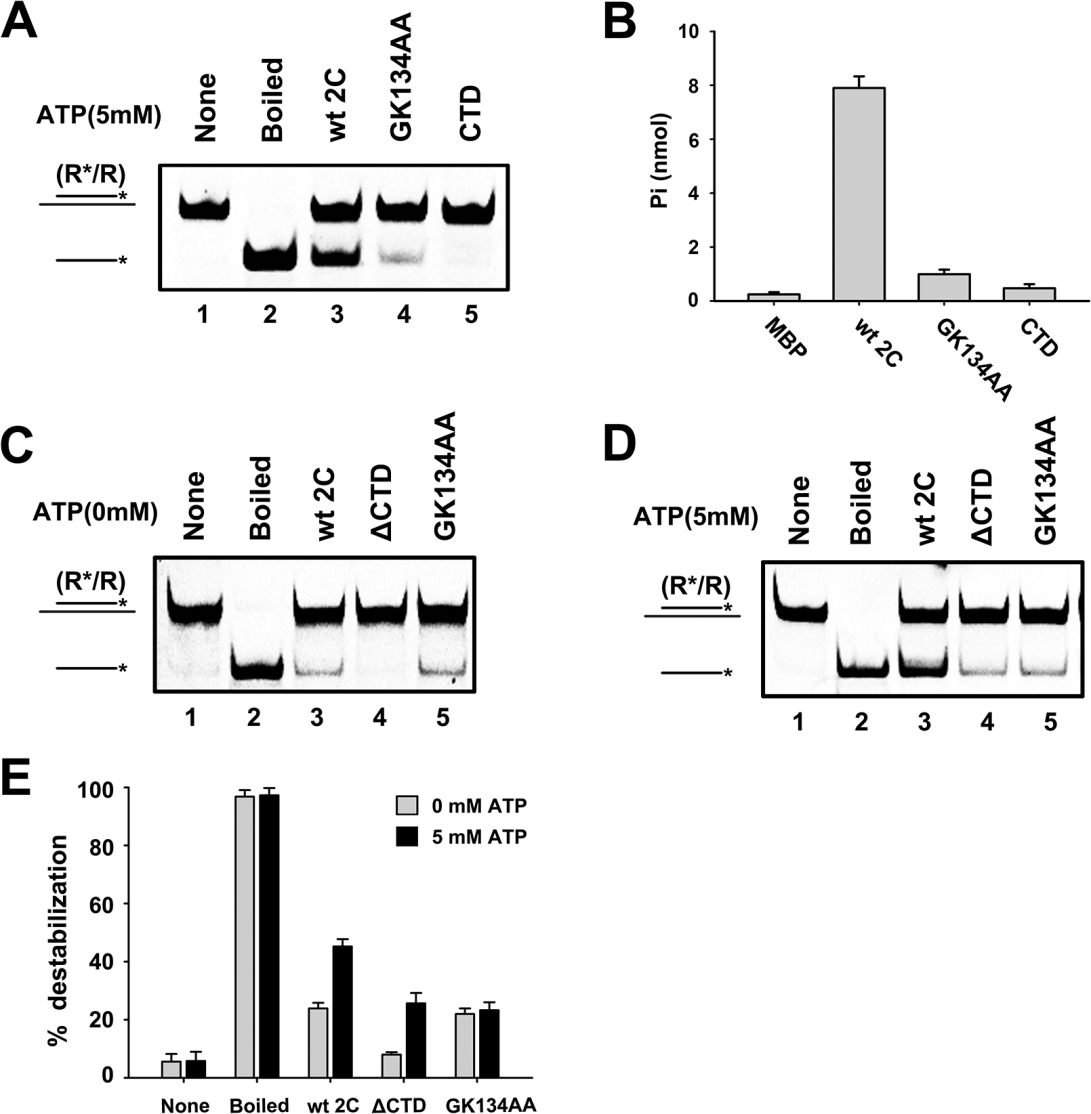
The CTD of 2C^ATPase^ is required for its RNA chaperone activity. **(A)** The standard RNA helix (0.1 pmol) was reacted with MBP-fusion 2C^ATPase^ Wt (lane 3), GK134AA mutant (lane 4), or a CTD fragment (lane 5). Native (lane 1) or boiled reaction mixture (lane 2) was used as a negative or positive control, respectively. **(B)** The ATPase activity of the indicated proteins was measured as nanomoles of released inorganic phosphate as indicated. MBP alone was used as the negative control. Error bars represent SD values from three separate experiments. **(C)** and **(D)** The standard RNA helix (0.1 pmol) was reacted with MBP-2C^ATPase^ Wt (lane 3), GK134AA mutant (lane 5), or ΔCTD (lane 4) in the absence (C) or presence (D) of 5 mM ATP as indicated. **(E)** The unwinding activities of Wt or indicated mutant 2C^ATPase^ in the absence or presence of 5 mM ATP were plotted as the percentage of the released RNA from the total RNA helix substrate. Error bars represent SD values from three separate experiments.

Our data showed that the GK134AA mutant of 2C^ATPase^ lost most ATPase activity (Fig 8B) but retained the RNA chaperone activity that is insensitive to ATP, as determined in these and previous experiments (Figs 5D, 8C and 8D); the motif C deletion (ΔSTN) of 2C^ATPase^ resulted in the loss of most helix unwinding activity (S6D Fig). Moreover, although 2C^ATPase^ CTD had no detectable helix unwinding activity and little ATPase activity (Fig 8A, lane 5; and Fig 8B), the loss of CTD resulted in the complete loss of helix unwinding in the absence of ATP (Fig 8C, lane 4), showing that the CTD is required for the RNA chaperone activity of 2C^ATPase^. Interestingly, in the presence of ATP, the helix unwinding activity of 2C^ATPase^ΔCTD was partially restored (Fig 8D, lane 4), indicating that the CTD plays an important but nonessential role in the helicase activity of 2C^ATPase^, probably by maintaining the proper structure of the 2C^ATPase^ helicase core domain.

### EV71 2C^ATPase^ facilitates enteroviral (+)RNA synthesis *in vitro*

**Fig 9:**
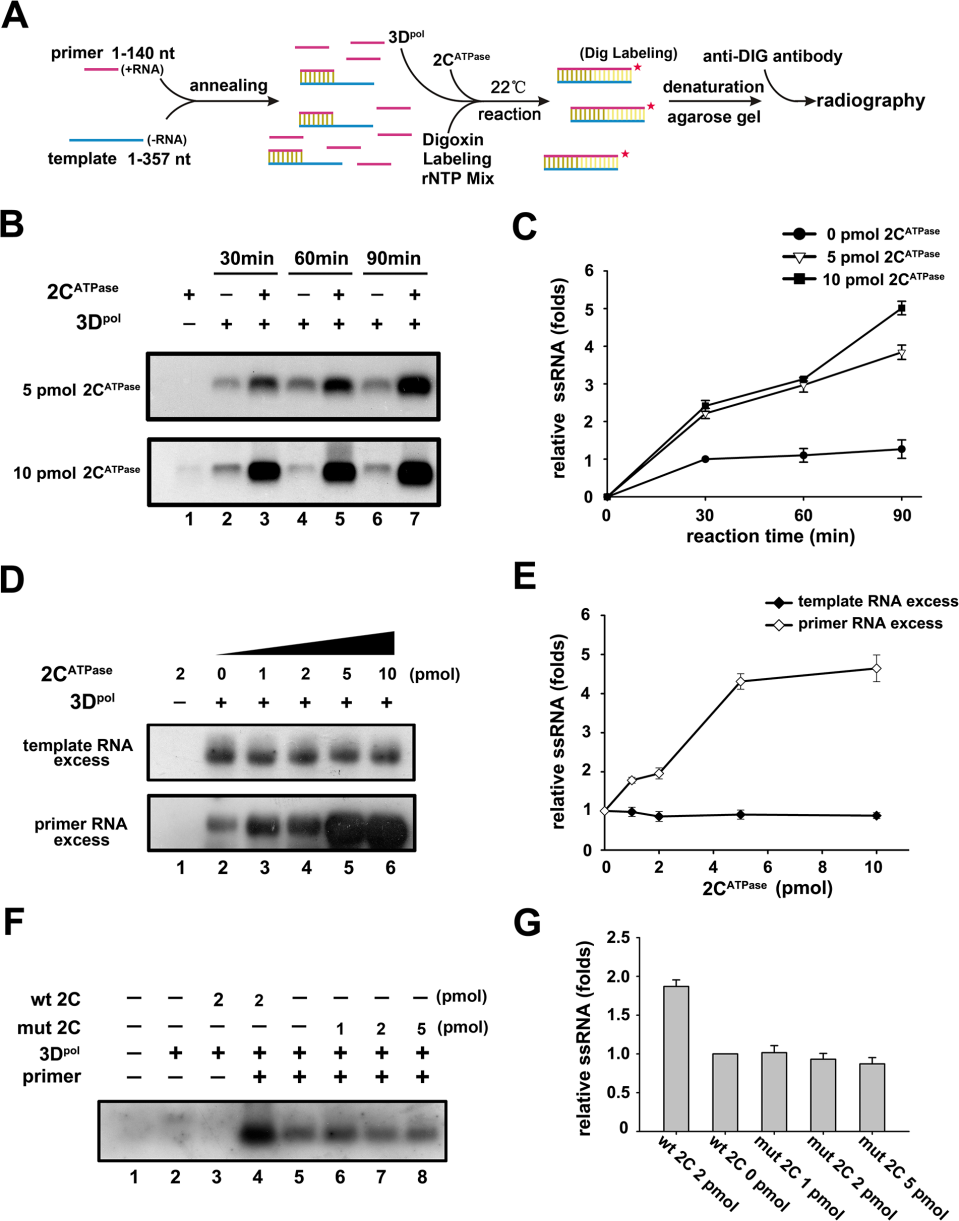
2C^ATPase^ facilitates 3D^pol^-mediated enteroviral RNA synthesis *in vitro*. **(A)** Schematic of the experimental procedures. **(B)** The *in vitro* transcribed EV71 3′-end (-)RNA template (10 pmol) and an excessive amount of primers (50 pmol) were preannealed and reacted with recombinant His-tagged EV71 3D^pol^ and DIG RNA labeling mix in the absence or presence of 5 pmol (upper panel) or 10 pmol (lower panel) MBP-2C^ATPase^ at 22°C for 30, 60, and 90 min as indicated. The reaction products were analyzed via electrophoresis on a denaturing formaldehyde-agarose gel. **(C)** The synthesized (+)RNA (DIG-labeled) products from the experiments in (B) were measured via Bio-Rad Quantity One software, and the relative RNA production was determined by comparing the RNA product level in the presence of the indicated amount of MBP-2C^ATPase^ at each time point with the RNA product level in the absence of MBP-2C^ATPase^ at 30 min. **(D)** The *in vitro* transcribed (-)RNA template and primers were preannealed and reacted with recombinant 3D^pol^ and DIG RNA labeling mix in the absence or presence of increasing amounts (1–10 pmol) of MBP-2C^ATPase^ at 22°C for 60 min as indicated. Upper panel: the amount of template (50 pmol) exceeded that of primer (10 pmol); lower panel: the amount of primer (50 pmol) exceeded that of template (10 pmol). **(E)** The synthesized (+)RNA products from the experiments in (C) were measured as in (B). Under either template- or primer-excessive conditions, the relative RNA production was determined by comparing the RNA product level in the presence of the indicated amount of MBP-2C^ATPase^ with the RNA product level in the absence of MBP-2C^ATPase^. **(F)** The RdRP reactions were conducted as in (B) under different conditions as indicated. **(G)** The synthesized (+)RNA products from the experiments in (F) were measured as in (B). Under each condition, the relative RNA production was determined by comparing the RNA product level in the presence of the indicated protein with the RNA product level in the absence of MBP-2C^ATPase^ (F, lane 5). For (C, E, and G), error bars represent SD values from three separate experiments.

After determining the RNA remodeling activity of 2C^ATPase^, we sought to evaluate its potential role in enteroviral RNA replication. It has been proposed that during vRNA replication of (+)RNA viruses, replicative intermediate double-stranded RNA must be unwound [45]. To assess the potential role of 2C^ATPase^ in enteroviral RNA replication, we first incubated purified His_6_-tagged 3D^pol^, the EV71 RdRP, with the (-)RNA template [i.e., we transcribed *in vitro* the 3′-end 1–357 nts of EV71 (-)RNA], and with an excessive amount (at a ratio of 5:1) of primer RNA, which was a long oligonucleotide and was pre-annealed to the template in the presence or absence of 2C^ATPase^ (Fig 9A). Of note, the RdRP reactions were conducted at 22°C as previously described [46], and at this temperature, EV71 2C^ATPase^ was still able to hydrolyze ATP and unwind the RNA helix, although both activities were moderately lower at 22°C than at 37°C (S4A and S4C Fig). Our data showed that the presence of 2C^ATPase^ significantly promoted the synthesis of (+)RNA strands from the (-)RNA template by 3D^pol^, and the promoting effect of 2C^ATPase^ was dose dependent (Fig 9B and 9C; Fig 9D, lower panel; and Fig 9E). Interestingly, when the amount of primer RNA was less than that of the (-)RNA template (at a ratio of 1:5), the presence of increasing amounts of 2C^ATPase^ was unable to promote (+)RNA synthesis (Fig 9D, upper panel; and Fig 9E), indicating that the major role of the 2C^ATPase^ helicase activity is not to directly enhance the RdRP activity of 3D^pol^ or smoothen the template. Of note, when the primer was absent, 3D^pol^ was unable to mediate RdRP or elongation reaction in the presence or absence of 2C^ATPase^ (Fig 9F, lanes 2 and 3). In addition, the GK134AA mutant of 2C^ATPase^ failed to promote (+)RNA synthesis as expected (Fig 9F, lanes 6–8; and Fig 9G). Altogether, our results show that 2C^ATPase^ can facilitate the 3D^pol^-mediated production of enteroviral RNA strands *in vitro* via promoting the efficiency to re-utilize the RNA template.

### The RNA helicase activity of 2C^ATPase^ is critical for the RNA replication and viability of EV71

**Fig 10:**
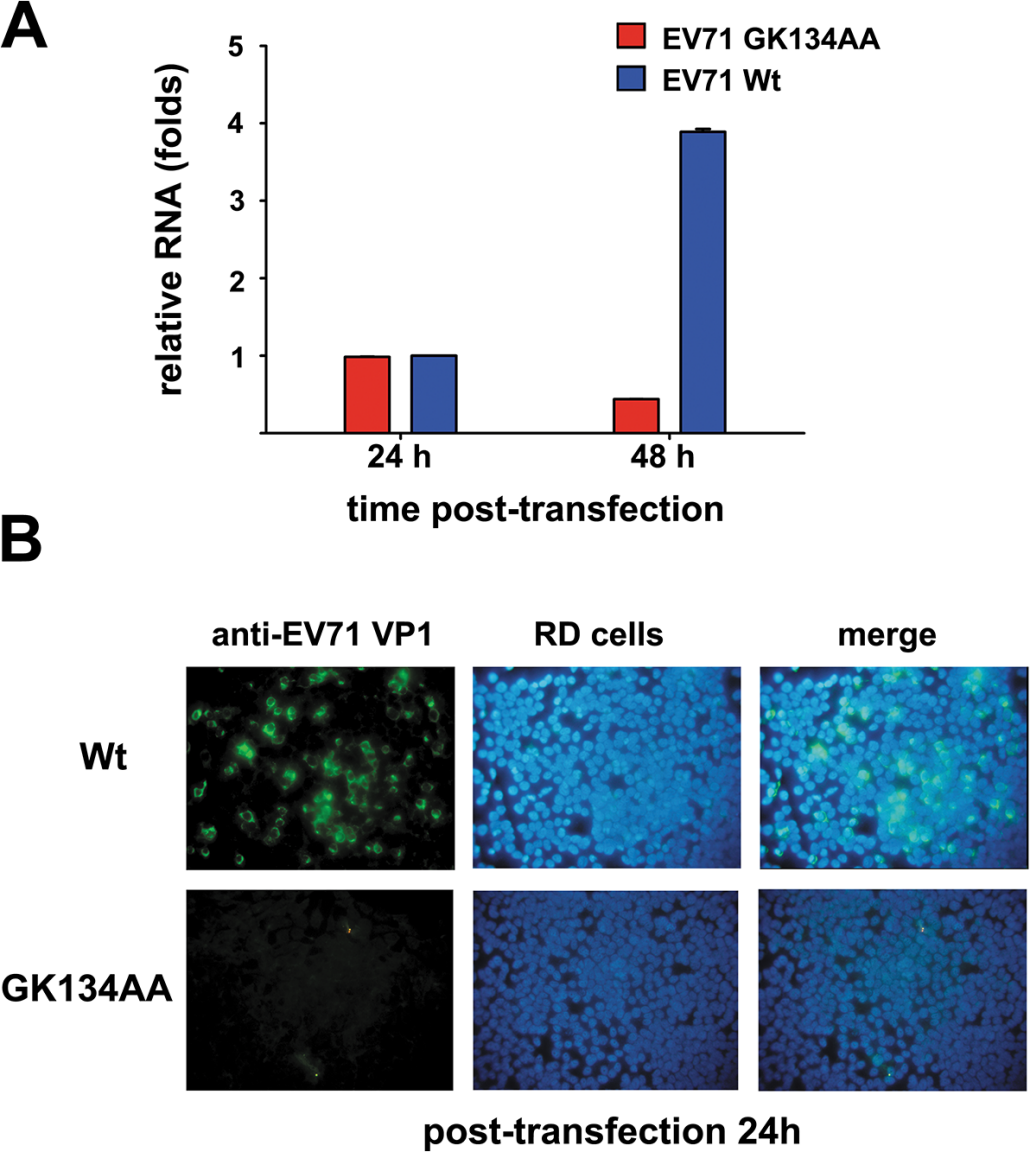
Effect of 2C^ATPase^ helicase-defective GK134AA mutation on EV71 RNA synthesis and viability. **(A)** Wt and GK134AA mutant EV71 RNA transcripts were transfected into human RD cells. The total RNA from differently transfected cells were exacted, and the levels of EV71 RNA were measured via qRT-PCR 24 and 48 hours post-transfection (h.p.t). For either Wt or mutant EV71 transcript, the relative EV71 RNA production 48 h.p.t. was determined by comparing the RNA product level at 48 h.p.t. with that at 24 h.p.t. Error bars represent SD, *n* = 3. **(B)** The virus production of either Wt (upper panel) or GK134AA mutant (lower panel) EV71 transcript in RD cells 24 h.p.t. was detected via immunofluorescent staining of EV71 VP1 with anti-VP1 antibody (green). The cell nuclei were stained with DAPI (blue). The merged image represents the digital superimposition of green and blue signals.

It has been previously reported that the GK-to-AA mutation in the motif A of poliovirus 2C^ATPase^ resulted in the inhibition of virus growth [47]. To determine whether the loss of the helicase activity of 2C^ATPase^ has any consequence on the viral life cycle of EV71, we introduced the GK134AA mutation, which disrupts the ATPase and helicase activities, in the 2C^ATPase^ coding region of the infectious clone of EV71. Then, Wt and mutant EV71 RNA transcripts were transfected into human rhabdomyosarcoma (RD) cells. RNA replication was measured via quantitative reverse transcription polymerase chain reaction (qRT-PCR) 24 and 48 hours post-transfection, and virus production was detected via immunofluorescent staining of EV71 VP1 in cells. Strikingly, compared to Wt virus, the GK134AA mutation resulted in the loss of EV71 RNA replication (Fig 10A) and viability (Fig 10B), suggesting that the GK motif and the RNA helicase activity of 2C^ATPase^ are critical for enteroviral RNA replication and viability.

### CAV16 2C^ATPase^ also possesses RNA helicase and chaperone activities

**Fig 11:**
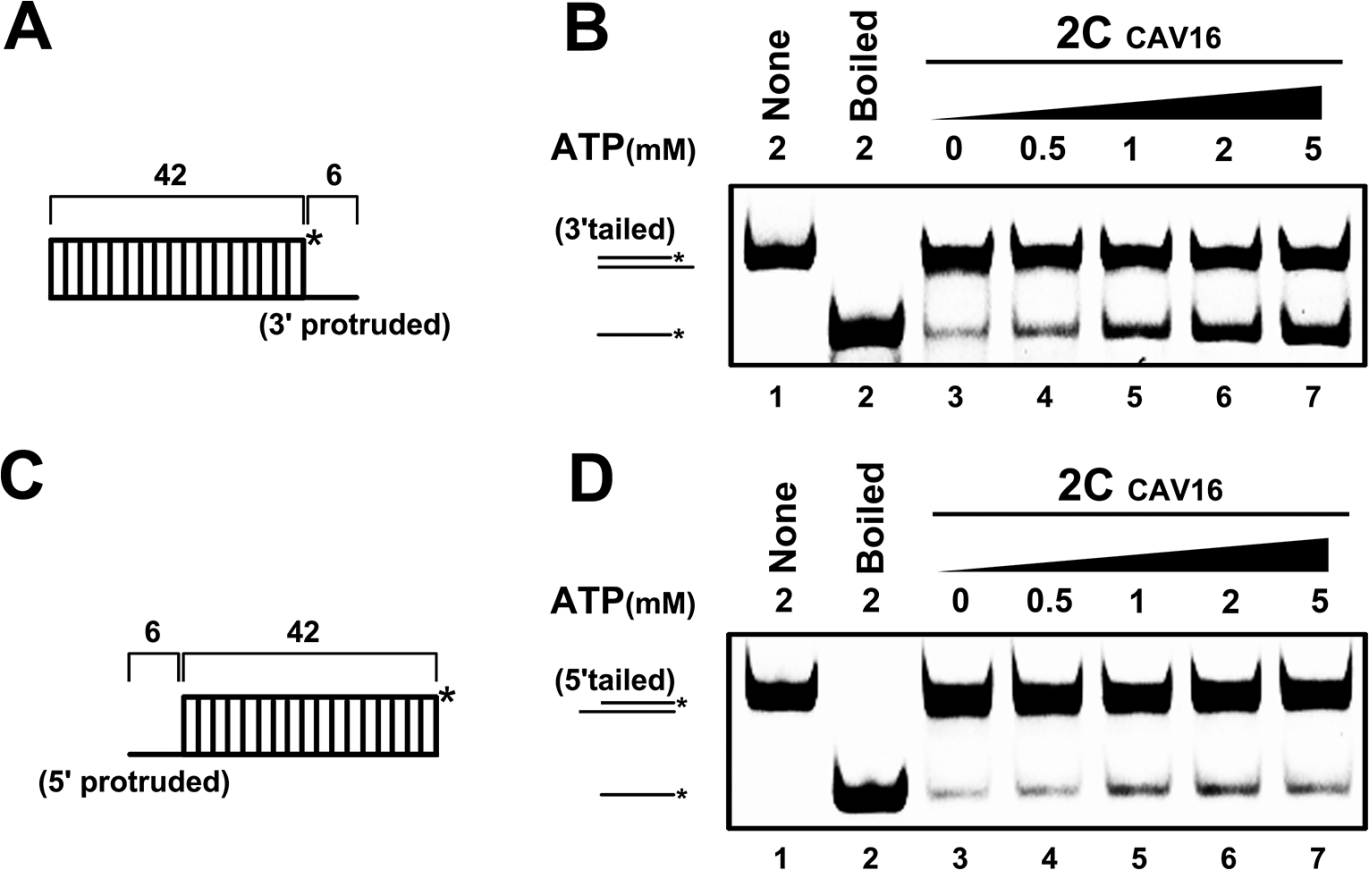
CAV16 2C^ATPase^ also contains both RNA helicase and chaperone activities. **(A)** Schematic illustration of the RNA helix substrate with the 3′ single-stranded protrusion. **(B)** The 3′-protruded RNA helix (0.1 pmol) was reacted with MBP-fusion CAV16 2C^ATPase^ (20 pmol) in the absence (lane 3) or presence (lanes 4–7) of increasing concentrations of ATP as indicated. **(C)** Schematic illustration of the RNA helix substrate with the 5′ single-stranded protrusion. **(D)** The 5′-protruded RNA helix (0.1 pmol) was reacted with MBP-fusion CAV16 2C^ATPase^ (20 pmol) in the absence (lane 3) or presence (lanes 4–7) of increasing concentrations of ATP as indicated. Asterisks indicate the HEX-labeled strands.

After determining that EV71 2C^ATPase^ possesses both RNA helicase and chaperone activities, we sought to determine whether 2C^ATPase^ encoded by another enterovirus exhibits similar activities. To this end, 2C^ATPase^ of CAV16 was eukaryotically expressed and then subjected to the helix unwinding assays using either a 3′- or 5′-protruded helix substrate. Exactly like EV71 2C^ATPase^ (Fig 5), CAV16 2C^ATPase^ unwound the 3′-protruded helix in an ATP-dependent manner (Fig 11A and 11B), whereas the unwinding of the 5′-protruded helix by this protein was ATP independent (Fig 11C and 11D). These data show that the ATP-dependent RNA helicase and ATP-independent RNA chaperone activities of 2C^ATPase^ are also conserved in CAV16, another enterovirus.

## Discussion

As the most conserved nonstructural protein among all enteroviruses and even picornaviruses, 2C^ATPase^ has been reported to participate in diverse processes critical for the enteroviral life cycle. Although this protein has long been predicted as a putative SF3 helicase on the basis of its AAA+ ATPase activity and conserved SF3 motifs, its potential role in viral RNA remodeling and replication remains elusive. Previous studies have shown that the ATPase activity of poliovirus 2C^ATPase^ could be inhibited by GnHCl. This compound is a potent inhibitor of enteroviral RNA replication *in vivo*, which has been reported to inhibit poliovirus (-)-vRNA synthesis and the uridylylation of the protein primer VPg [38,39], suggesting that the ATPase activity of 2C^ATPase^ is critical for enteroviral RNA replication. Here we report that protein 2C^ATPase^ of EV71 possesses a nonconventional RNA remodeling activity that can function as both an ATP-dependent RNA helicase and an ATP-independent RNA chaperone and facilitates EV71 RdRP-mediated vRNA synthesis from vRNA template *in vitro*. The mutation of GK134 to AA, which disrupts 2C^ATPase^ helicase activity, mostly abolished EV71 RNA replication and virus production. These data indicate that 2C^ATPase^-mediated RNA remodeling is pivotal for the enteroviral life cycle. Moreover, our results show that the RNA remodeling activities of 2C^ATPase^ are also conserved in CAV16.

Although 2C^ATPase^ contains conserved motifs typical of SF3 helicases, previous attempts to identify its helicase activity were unsuccessful [27], leading us to ask why the RNA helicase/chaperoning activity of 2C^ATPase^ can be detected in this study. It is noteworthy that the recombinant poliovirus 2C^ATPase^ protein used in the previous assay was produced in *E*. *coil*, while the EV71 and CAV16 2C^ATPase^ proteins used here were expressed using an eukaryotic system (baculovirus). Interestingly, we found that although both the prokaryotically and eukaryotically expressed 2C^ATPase^ proteins possessed similar ATPase activity (S7 Fig), only the eukaryotically expressed 2C^ATPase^ protein possessed the RNA duplex unwinding activity (Fig 1F). Moreover, the eukaryotically expressed 2C^ATPase^ showed an apparently higher molecular mass than the prokaryotically expressed one (Fig 1E), implying that the former protein contains some post-translational modifications that potentially play a critical role(s) in the helicase activity of 2C^ATPase^. Future research would uncover the potential post-translational modifications of 2C^ATPase^.

Although they have mainly been characterized *in vitro*, the RNA remodeling activities of virus-encoded proteins are generally believed to play critical roles in the viral life cycle [48]. The RNA genomes contain multiple *cis*-acting elements, including the 5′ cloverleaf, 3′ UTR-poly(A), internal origin of replication (*oriI* or *cre*) [49,50], internal ribosomal entry site [51,52], and the cloverleaf at the 3′-end of (-)-vRNA [53–55]. These highly structured RNA elements play indispensable roles in the replication, translation, and encapsidation of enteroviral RNAs [23,24,54,56] and probably require the RNA chaperoning activity of 2C^ATPase^ to facilitate their proper folding and refolding. On the other hand, during the RNA replication of RNA viruses, replicative intermediate dsRNA must be unwound, thereby allowing the efficient recycling of vRNA template for progeny vRNA synthesis. It is very likely that the RNA helicase activity of 2C^ATPase^ plays such a dsRNA unwinding role during enteroviral RNA replication, which is consistent with our observations that 2C^ATPase^ can facilitate RdRP-mediated enteroviral RNA synthesis *in vitro* by promoting the recycling of vRNA template (Fig 9), and disrupting 2C^ATPase^ RNA helicase activity in EV71 resulted in an almost complete loss of enteroviral RNA replication and virus production in cells (Fig 10). Interestingly, although RNA chaperones can also unwind/destabilize RNA duplexes, RNA chaperones are normally not considered to be involved in unwinding viral replicative dsRNA, as they usually destabilize short base pairs or hairpins within structured RNA elements. Indeed, Banerjee *et al*. reported that poliovirus 2C^ATPase^ can bind the 3′-end cloverleaf structure of (-)-vRNA [15], implying a role of 2C^ATPase^ in this structured element. In addition, our finding that disrupting 2C^ATPase^ helicase activity but not chaperone activity mostly abolished enteroviral RNA replication and viability strongly suggests that the functions associated with RNA helicase and chaperone play different roles in the same RNA molecule (vRNA) and cannot replace each other.

We previously reported that 2C^ATPase^ from EoV, an insect picorna-like virus belonging to the family *Iflaviridae*, possesses an ATP-independent RNA chaperone activity but not helicase activity [30]. Interestingly, although EoV 2C^ATPase^ is predicted to contain conserved SF3 motifs A, B, and C, the GK-to-AA mutation in EoV 2C^ATPase^ motif A did not abolish but instead increased its ATPase activity [30]. This observation obviously contradicts the indispensable role of the motif A “GKS” in the ATPase and helicase activities of defined SF3 helicases, including EV71 2C^ATPase^ (Fig 8), confirming that EoV 2C^ATPase^ is not a SF3 helicase. And the functional disparity between these two proteins is probably due to the long-time evolution and adaptation of iflavirus and enterovirus in different types of hosts, insects, and mammals.

Interestingly, enterovirus 2C^ATPase^ is not the only RNA remodeling protein encoded by enteroviruses. Previous studies by DeStefano and colleagues showed that poliovirus 3AB is an RNA chaperone [42,57]; moreover, our previous report confirmed that EV71 3AB also contains *in vitro* RNA chaperone activity [58]. In addition, poliovirus 3D^pol^ has been reported to display unwindase activity that does not require ATP hydrolysis but does require an RNA chain elongation reaction [59]. It is plausible that 2C^ATPase^ and 3D^pol^ work synergistically to unwind nascently synthesized vRNA strands from template. On the other hand, the chaperone activities of 2C^ATPase^ and 3AB may function collaboratively or separately on different structured *cis*-acting elements of enteroviral RNA. However, owing to technical limitations, it is almost infeasible to determine the exact role of RNA helicase or chaperoning activity in the structures and functionalities of RNA molecules in cells or *in vivo*. Future studies may overcome these technical barriers and provide a mechanistic view of how RNA remodeling proteins function in cells.

RNA helicases and ATP-independent RNA chaperones are recognized as the two distinct classes of RNA remodelers. RNA helicases participate in most ATP-dependent rearrangement of structured RNAs and RNA-protein complexes [60,61]. On the other hand, RNA chaperones are able to unwind RNA duplexes and facilitate RNA strand annealing, thereby aiding the folding or refolding of RNA molecules into correct structures. The major difference between RNA helicase and chaperone is that the former needs the participation of ATP, but the later does not. Different from previously characterized RNA remodeling proteins, enteroviral 2C^ATPase^ can function as both a typical RNA helicase and an ATP-independent RNA chaperone. That a single protein can contain these two different types of RNA remodeling activities is intriguing. Our structural analyses of protein 2C^ATPase^ may provide some hints. Protein 2C^ATPase^ contains a 137-a.a. middle domain that includes the conserved SF3 signature A, B, and C motifs and is structurally similar to the helicase core domains of other SF3 viral helicases, indicating that the middle domain provides the basis of its RNA helicase activity. On the other hand, although RNA chaperones have been studied for decades, the mechanism(s) governing their ATP-independent remodeling activity remains elusive. A popular model proposed to explain the mechanism is the “entropy or disorder transfer”, in which certain intrinsic disordered or unstructured regions of RNA chaperones can transfer their disorder or entropy to RNA molecules, and such a transfer of disorder or entropy can destabilize misfolded RNAs and aids RNA refolding. Indeed, many RNA chaperones, such as HIV-1 Vif, Tat and nucleocapsid, flavivirus core protein, hantavirus N protein, and cypovirus VP5, have been predicted to contain multiple intrinsic disordered regions [40,48], providing support for this model. Structure modeling using ROBETTA indicated that the CTD of 2C^ATPase^ contains a highly unstructured or disordered coiled-coil region that links the helicase core domain and the CTD (Fig 1C). When the CTD was deleted from the protein, the truncation completely abolished the RNA chaperoning activity of 2C^ATPase^ (Fig 8C). The truncation of CTD reduced but did not abolish the RNA helicase activity (Fig 8D), suggesting that the presence of CTD is also important for the helicase activity, probably by affecting the protein's global conformation. Moreover, the CTD alone does not contain any RNA remodeling activity (Fig 8A), suggesting that the RNA chaperoning function of 2C^ATPase^ requires the RNA binding capacities of other 2C^ATPase^ domains. The coiled-coil regions and the middle core domains are highly conserved in enteroviruses and other picornaviruses, such as encephalomyocarditis virus (EMCV) and foot-mouth-disease virus (FMDV) (S8 Fig). On the basis of all these findings, we propose that the RNA helicase function of enteroviral 2C^ATPase^ relies on the conserved helicase motifs and ATPase activity, while specific structural characteristics, like the architecture of RNA binding and intrinsic disorder regions, are the basis of its RNA chaperoning function. Moreover, these findings provide evidence of the correlation between intrinsic disorder and RNA chaperone activity, which supports the “entropy transfer” model of RNA chaperones.

In conclusion, this study determined the RNA helicase and chaperoning activities associated with enterovirus 2C^ATPase^ and provides both *in vitro* and cellular evidence of their potential functions in enteroviral RNA replication. It also provides the first evidence that the two distinct types of RNA remodeling activities, RNA helicase and ATP-independent RNA chaperoning activities, can be integrated within one protein, introducing an extended view of RNA remodeling proteins. Altogether, these findings increase our understanding of enteroviruses and the two types of RNA remodeling activities.

## Materials and Methods

### Construction of recombinant baculoviruses

The generation of pFastBac HTB-MBP and pFastBac HTB-MBP-NS3_HCV_ have been described previously [30]. The cDNAs for EV71 2C^ATPase^ (GenBank Accession No. KC954662), CAV16 2C^ATPase^ (GenBank Accession No. KC117318) and EV71 2C^ATPase^ fragments were amplified by polymerase chain reaction (PCR) from the plasmid containing full-length EV71 cDNA, and cloned into the vector pFastBac HTB-MBP. Site-directed mutations were generated as previously described [30]. The primers used in this study are listed in Table. The resulting plasmids were subjected to Bac-to-Bac baculovirus system to express the recombinant proteins with an MBP fused at the N-terminal.

### Expression and purification of recombinant proteins

The expression and purification of MBP alone and MBP-fusion proteins were performed as previously described [30,40]. Briefly, Sf9 cells were infected with the recombinant baculoviruses and harvested at 72 h postinfection. Cell pellets were resuspended, lysed by sonication and subject to centrifugation for 30 min at 11 000 g to remove debris. The protein in the supernatant was purified using amylase affinity chromatography (New England BioLabs, Ipswich, MA) according to the manufacturer's protocol, and then concentrated using Amicon Ultra-15 filters (Millipore, Schwalbach, Germany). All proteins were quantified by the Bradford method and stored at -80°C in aliquots. Proteins were separated on 10% SDS-PAGE and visualized by Coomassie blue.

### Structural modeling analysis of enteroviral 2C^ATPase^

The 3D structure of EV71 2C^ATPase^ was modeled by submitting its amino acid sequence to the HMMSTR/Rosetta server [from Robetta, University of Washington (http://robetta.bakerlab.org/)] [31]. Five models were obtained, and the best one was chosen as a template based on its score, assessed by submitting it to the SWISS-MODEL Server [from Swiss Institute of Bioinformatics and the Biozentrum, University of Basel, Switzerland (http://swissmodel.expasy.org)]. The figure of the modeled EV71 2C^ATPase^ 3D structure and the structural alignment with AAV2 Rep40 (PDB ID code 1U0J) were drawn by PyMOL program 1.1 (DeLano Scientific LLC, South San Francisco, CA) from coordinate file.

### Preparation of oligonucleotide helix substrates

In brief, of the two strands, one was labeled at the 5′ end with hexachloro-fluorescein (HEX), and the other strand was unlabeled. HEX-labeled oligonucleotide strands were purchased from TaKaRa (Dalian, China). Unlabeled DNA strands were synthesized by Invitrogen, and unlabeled RNA strands were *in vitro* transcribed using T7 RNA polymerase (Promega, Madison, WI). The *in vitro* transcribed RNA strands were purified by Poly-Gel RNA Extraction Kit (Omega bio-tek, Guangzhou, China) according to the manufacturer's instruction. The two strands were mixed in a proper ratio, and annealed through heating and gradually cooling as previously described [30,40]. The standard RNA helix substrate with both 5′ and 3′ protrusions was annealed with RNA1 and RNA2, the R*/D substrate was annealed with RNA1 and DNA1, the D*/D substrate was annealed with DNA2 and DNA3, the D*/R substrate was annealed with DNA2 and RNA3, the 3′-protruded RNA helix was annealed with RNA1 and RNA4, the 5′-protruded RNA helix was annealed with RNA1 and RNA5, the blunt-ended substrate was annealed with RNA1 and RNA6, and the 49 matched bps substrate was annealed with RNA7 and RNA8. All oligonucleotides used in this study are listed in S2 Table.

### Nucleic acid helix unwinding and strand hybridization assays

The standard helix destabilizing assay was performed as previously described [40] with minor modifications. Briefly, 20 pmol of recombinant protein and 0.1 pmol of HEX-labeled helix substrate were added to a mixture containing 50 mM HEPES-KOH (pH 7.5), 2.5 mM MgCl_2_, and 2 mM Dithiothreitol (DTT), 0.01% bovine serum albumin (BSA), and 15 U RNasin (Promega). After incubation at 37°C for 60 min or indicated time, the reaction was terminated by adding proteinase K (final concentration of 1 μg/μl) and 5×loading buffer [100 mM Tris-HCl, 1% SDS, 50% glycerol, and bromophenol blue (pH 7.5)]. The mixtures were then electrophoresed on 15% native-PAGE gels, followed by scanning with a Typhoon 9200 imager (GE Healthcare, Piscataway, NJ). The RNA strand hybridization assay was performed as previously described [40]. The sequences of the stem-loop-structured RNA strands were indicated in Fig 7A. The samples were also resolved on 15% native-PAGE gels, followed by gel scanning a Typhoon 9200 imager (GE Healthcare).

### NTPase assay

NTPase activities were determined via measuring the released inorganic phosphate during NTP hydrolysis using a direct colorimetric assay as previously described [30]. All of the results given with this quantitative assay were averages of three repeated experiments.

### Ribozyme cleavage assay

The ribozyme RNA was synthesized from the *in vitro* transcription using T7 RNA polymerase, and the 5′-HEX-labeled substrate RNA was synthesized by TaKaRa. Indicated amount of protein was added in 10 μl reaction volumes containing 0.15 nM substrate, 0.3 nM ribozyme, 50 mM HEPES-KOH (pH 7.5), 2.5 mM MgCl_2_, and 2 mM DTT, 0.01% BSA, 20 U RNasin. The mixtures were incubated at 37°C for 30 min, and then treated by proteinase K (final concentration of 1 μg/μl) at 37°C for 15 min. The digestion reactions were precipitated with 20 μl isopropanol and 2 μg glycogen (-80°C, 30 min), and subjected to by centrifugation at 12 000 *g* for 15 min. The precipitates were dissolved in formamide and electrophoresed on 15% acrylamide-7 M urea gels, followed by scanning with a Typhoon 9200 imager (GE Healthcare).

### *In vitro* RdRP assays

The RNA primer and template were *in vitro* transcribed by T7 RNA polymerase as described above. The primer RNA strand contains the 1–140 nts of the 5′ end of EV71 (+)RNA, and the (-)-vRNA template contains the sequence complementary with the 1–357 nts of 5′ end of EV71 (+)RNA. The transcribed products were purified and quantified as described above. Then, the primer RNA was pre-annealed to the template. The EV71 3D^pol^ gene was cloned into the vector pET26b-Ub plasmid, and the protein was purified as the C-terminal His×6 tag fusion protein via a nickel-charged HisTrap HP column (GE Healthcare) as previously described [62]. The RdRP reactions were carried out in a total volume of 10 μl at 22°C as previously described [46]. As previously described [63], the RdRP reactions were carried out in the reaction mixture containing 500 nM 3D^pol^ in 50 mM HEPES-KOH (pH 7.0), 12.5 mM KCl, 5 mM MgCl_2_, 5 mM DTT, 20 U RNasin (Promega), and 0.25 mM Digoxin (DIG) RNA Labeling Mix (Roche) with total volume of 10 μl at 22°C. The reaction mixtures were supplemented with 10 μl 2×loading buffer (20% 10×MOPS running buffer, 10% glycerol, 50% formamide, 20% formaldehyde and 0.05% bromophenol blue), and then denatured at 65°C for 10 min. The samples were electrophoresed on 1.5% agarose-formaldehyde denaturing gels, and transferred onto N^+^ nylon membranes (Roche). The membranes were incubated with anti-DIG-alkaline phosphatase antibody (Roche), followed by incubation with CDP-Star (Roche) at 37°C for 10 min. The signals were detected by radiography on X-ray film (FujiFilm, Tokyo, Japan). All oligonucleotides used for *in vitro* transcription are listed in S3 Table.

### Cells

Human rhabdomyosarcoma (RD) cells were obtained from the American Type Culture Collection (ATCC), and cultured in Dulbecco's modified Eagle's medium (DMEM, Gibco) containing 10% fetal bovine serum (FBS, Gibco), 100 units of penicillin and 100 mg/ml of streptomycin. The transfected or infected RD cells were maintained in DMEM containing 2% FBS.

### EV71 RNA transcription and transfection

The wild type and mutant plasmids containing full-length EV71 cDNA were linearized with *Mlu* I digestion. 2 μg of each purified linear plasmid was used as the template to *in vitro* transcribe EV71 RNA using the SP6 RiboMAX Large Scale Production System (Promega) according to the manufacturer's protocols. The RNA transcript was transfected into 80%-90% confluent RD cells using Lipofectamine 2000 (Invitrogen). 24–48 h post-transfection, the total RNA from transfected RD cells was extracted using RNeasy Mini Kit (QIAGEN, GmBH, Germany), and stored at -80°C.

### One-step Quantitative Real-Time RT-PCR (qRT-PCR)

The amount of viral RNA was quantified by using One Step PrimeScript RT-PCR Kit (Takara, Dalian) according to the manufacturer's protocol as previous described [64] with EV71 specific premiers (Fwd, 5′-GGCCATTTATGTGGGTAACTTTAGA-3′; Rev, 5′-CGGGCAATCGTGTCACAAC-3′) and probe (5′ FAM-AAGACAGCTCTCGCGACTTGCTCGTG-BQH1 3′).

### Indirect immunofluorescence assay

RD cells at 90% confluence on glass slides were transfected with indicated EV71 transcript. The cells were fixed at 24 h post-transfection with pre-cooled acetone at -20°C for 30min, followed by washing in PBS for 3 times and incubation with mouse anti-EV71 VP1 monoclonal antibody (Chemicon International, Madison, WI) for 60 min at 37°C. The cells were then washed in PBS and incubated with Alexa Fluor 488-labeled goat anti-mouse IgG (Invitrogen) for another 45 min. Cells were incubated with DAPI at room temperature for 5 min to stain the nuclei. Finally, the cells were rinsed again with PBS and visualized under a fluorescent microscope (Olympus, Tokyo, Japan).

## Supporting Information

S1 Fig Silver staining and mass spec analysis of recombinant MBP-2C^ATPase^. MBP-fusion EV71 2C^ATPase^ was expressed using eukaryotic (baculovirus) system. The purified recombinant proteins were subjected to 10% SDS-PAGE followed by silver staining. M, molecular mass marker.
(TIF)

S2 Fig Size exclusion chromatography of recombinant MBP-2C^ATPase^. **(A)** Elution profile of purified MBP-2C^ATPase^ from a Superdex 200 increase 10/300 GL column. Protein elution was followed by UV detection at 280 nm. X axis represents the elution volume (in ml). The major elution peak corresponds to a molecular mass of ~600 kDa, which is estimated according to the manufacturer's instruction. **(B)** SDS-PAGE and silver staining of the eluted protein in the major peak.
(TIF)

S3 Fig Characterization of the RNA helix unwinding activity of 2C^ATPase^. 0.1 pmol RNA helix substrate with 49 (lanes 1–3) or 42 (lanes 4–6) complementary base pairs was reacted with MBP-2C^ATPase^ (20 pmol). Lanes 1 and 4, reaction mixture without 2C^ATPase^ addition; lanes 2 and 5, boiled reaction mixture without 2C^ATPase^. Asterisks indicate the HEX-labeled strand.
(TIF)

S4 Fig Biochemical characterization for the NTPase and RNA helix unwinding activities of EV71 2C^ATPase^. **(A)** The NTPase activity of MBP-2C^ATPase^ on each NTP in the absence or presence of 100 pmol ssRNA was measured at 37°C as nanomoles of released inorganic phosphate as indicated. In addition, the ATPase activity of MBP-2C^ATPase^ was measured at 22°C as indicated. MBP alone was used as the negative control. Error bars represent SD values from three separate experiments. **(B)** The unwinding assays of HCV NS3 using the standard helix substrate were performed in the absence or presence of 5 mM ATP or AMP-PNP as indicated. **(C)** The unwinding assays of MBP-2C^ATPase^ using the standard helix substrate were performed at 22°C or 37°C as indicated. Boiled MBP-2C^ATPase^ or no protein supplementation was used as negative control. **(D)** The standard RNA helix (0.1pmol) was reacted with MBP-2C^ATPase^ (20 pmol) in the presence of indicated divalent metallic ions (2.5 mM). **(E)** The standard RNA helix was reacted with 2C^ATPase^ (20 pmol) in varying concentrations of Zn^2+^. **(F)** The standard RNA helix was reacted with 2C^ATPase^ (20 pmol) at the indicated pH. For (B-F), asterisks indicate the HEX-labeled strand.
(TIF)

S5 Fig The effect of different NTP on the helix unwinding activity of 2C^ATPase^. The standard RNA helix was reacted with 2C^ATPase^ (20 pmol) in the absence (lane 3) or presence of 5 mM indicated NTP (lanes 4–7). Asterisks indicate the HEX-labeled strand.
(TIF)

S6 Fig (A-C) The modeled structures of EV71 2C^ATPase^ full-length (A), and CTD-truncated (ΔCTD) (B) and CTD fragments (C), as predicted using the HMMSTR/Rosetta server. The sites of “GK” in helicase/ATPase motif A and “STN” in helicase motif C are indicated as yellow and green, respectively. **(D)** The standard RNA helix (0.1 pmol) was reacted with MBP-2C^ATPase^ wt (lane 4) or ΔSTN mutant (lane 3) in the presence of 5 mM ATP. Asterisks indicate the HEX-labeled strand. **(E)** Elution profile of purified MBP-2C^ATPase^ΔCTD from a Superdex 200 increase 10/300 GL column. Protein elution was followed by UV detection at 280 nm. X axis represents the elution volume (in ml).
(TIF)

S7 Fig The ATPase activity of prokaryotically and eukaryotically expressed MBP-2C^ATPase^. The ATPase activity of indicated proteins was measured at 37°C as nanomoles of released inorganic phosphate. MBP alone was used as the negative control. Error bars represent SD values from three separate experiments.
(TIF)

S8 Fig Sequence alignments of 2C^ATPase^ proteins from different picornaviruses. **(A)** The sequence alignment of the linker region between the HC domain and CTD of 2C^ATPase^ proteins from indicated picornaviruses. **(B)** The sequence alignment of the HC domains of 2C^ATPase^ proteins from indicated picornaviruses. EV71, enterovirus 71, genus *Enterovirus*; CAV16, coxsackie A virus 16, genus *Enterovirus*; PV, poliovirus, genus *Enterovirus*; EMCV, encephalomyocarditis virus, genus *Cardiovirus*; FMDV, foot-and-mouth disease virus, genus *Aphthovirus*. The alignments were conducted using ClustalX2. The conserved motifs A, B, and C for SF3 helicases are indicated.
(TIF)

S1 Table Mass spectrometry analysis of purified MBP-2C^ATPase^. (XLSX)

S2 Table List of oligonucleotides. (DOC)

S3 Table List of primers. (DOC)

S1 Text Supplementary materials and methods, and references. (DOCX)
