# Supplementary figures and images for: Human Enterovirus Nonstructural Protein 2CATPase Functions as Both an RNA Helicase and ATP-Independent RNA Chaperone
Source: PLoS Pathog. 2015 Jul 28;11(7):e1005067. doi: 10.1371/journal.ppat.1005067 (PMC4517893; doi:10.1371/journal.ppat.1005067)

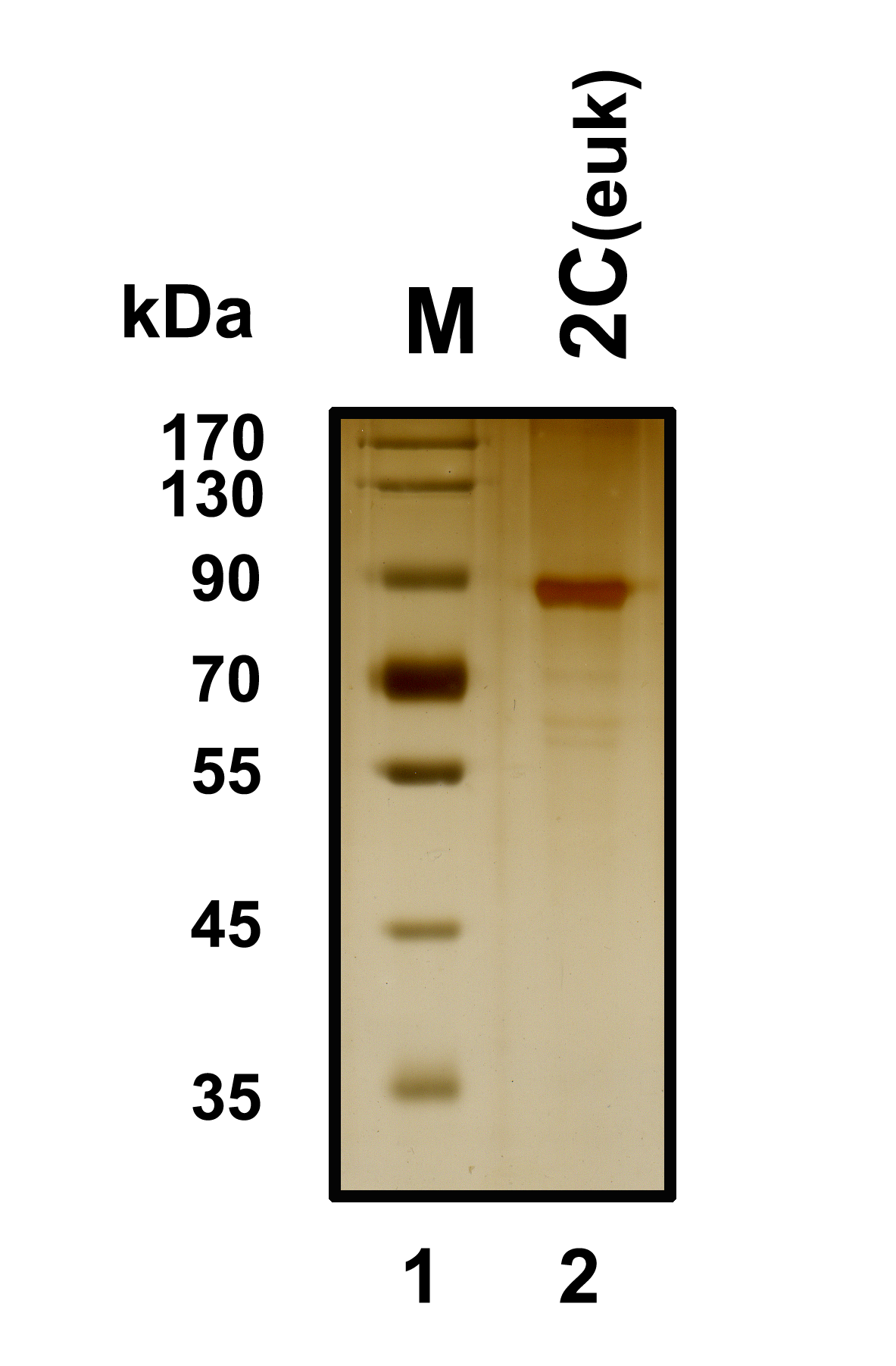

Supplement: S1 Fig — MBP-fusion EV71 2CATPase was expressed using eukaryotic (baculovirus) system. The purified recombinant proteins were subjected to 10% SDS-PAGE followed by silver staining. M, molecular mass marker. (TIF) [file ppat.1005067.s001.tif]

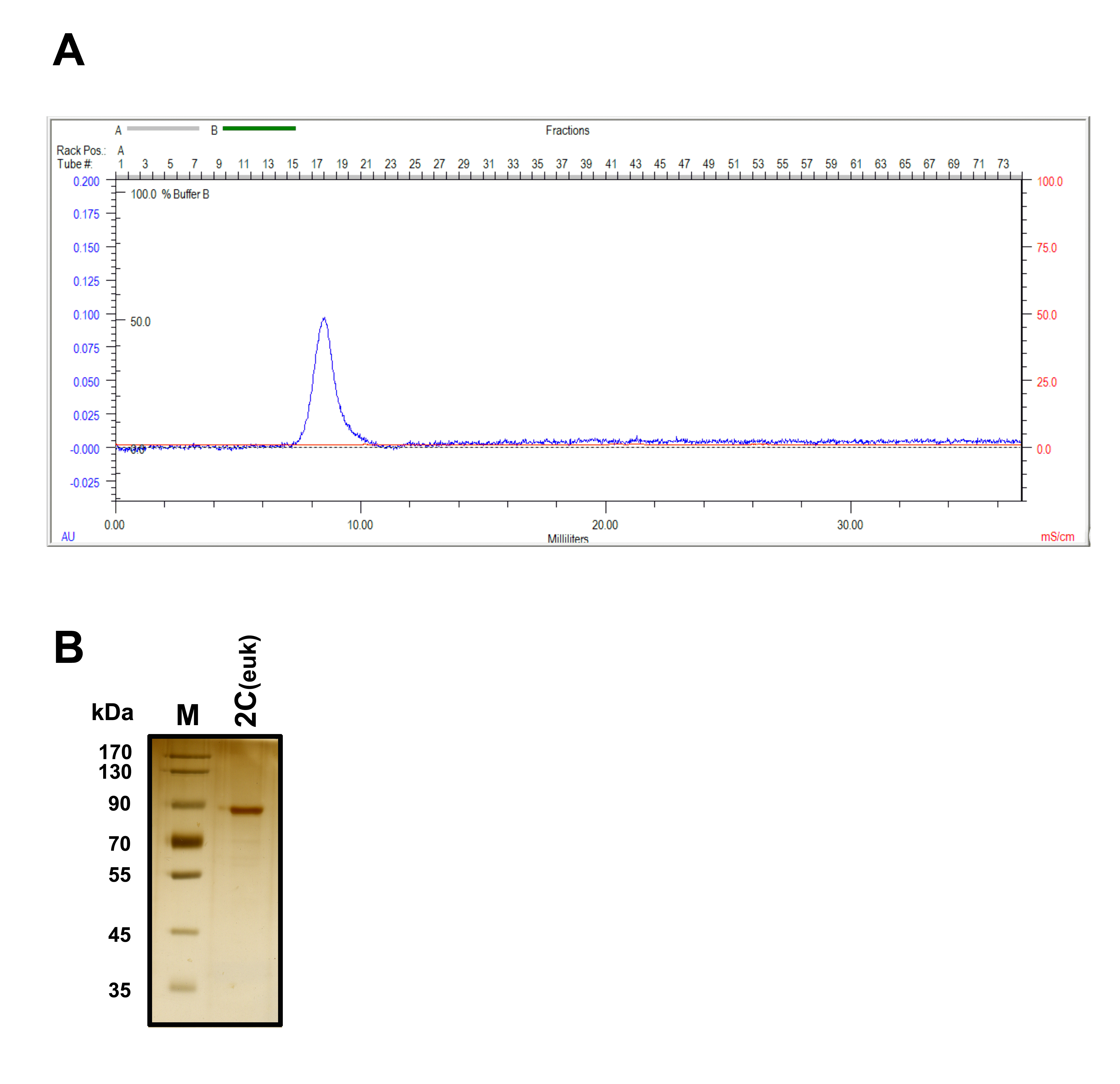

Supplement: S2 Fig — (A) Elution profile of purified MBP-2CATPase from a Superdex 200 increase 10/300 GL column. Protein elution was followed by UV detection at 280 nm. X axis represents the elution volume (in ml). The major elution peak corresponds to a molecular mass of ~600 kDa, which is estimated according to the manufacturer's instruction. (B) SDS-PAGE and silver staining of the eluted protein in the major peak. (TIF) [file ppat.1005067.s002.tif]

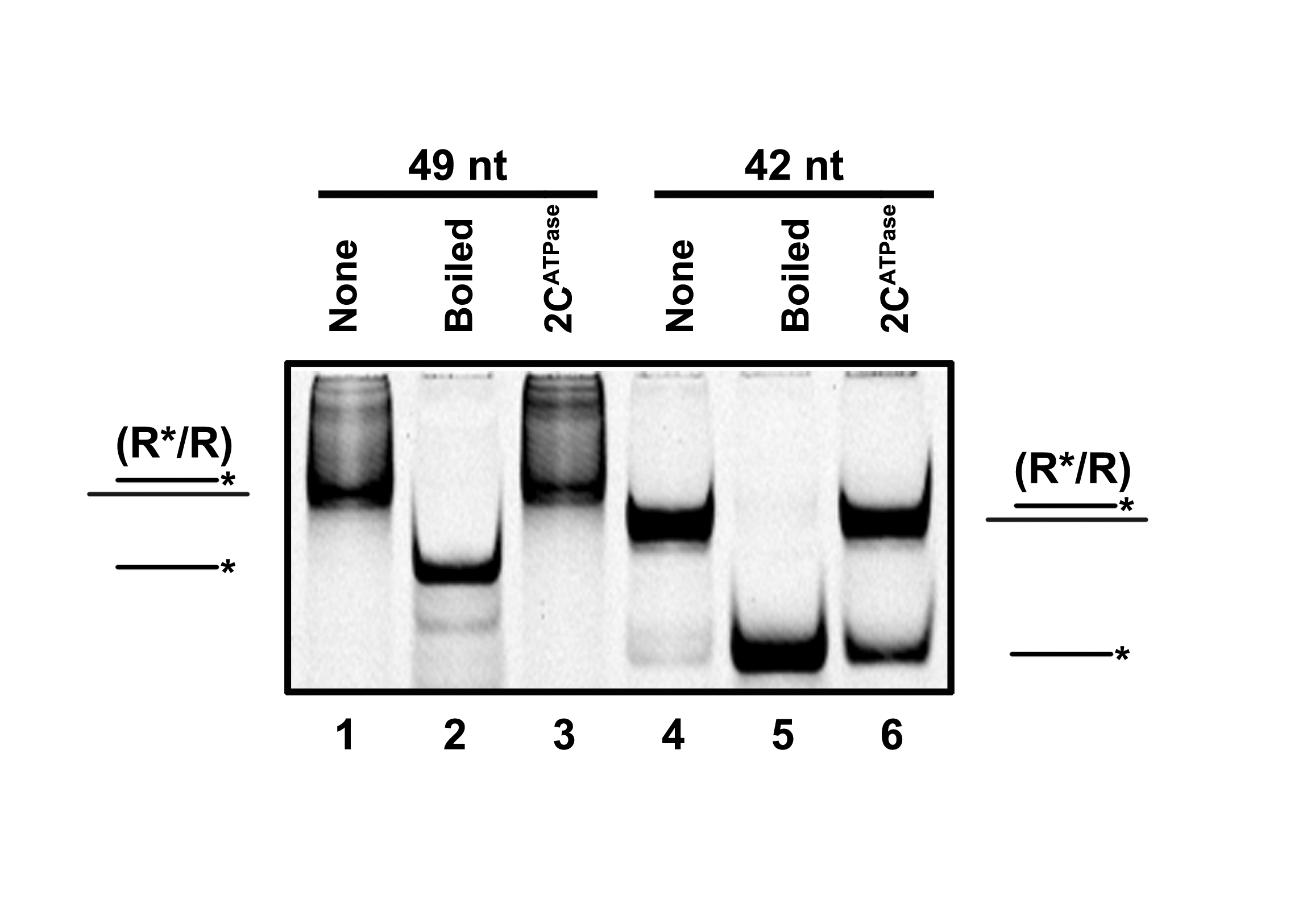

Supplement: S3 Fig — 0.1 pmol RNA helix substrate with 49 (lanes 1–3) or 42 (lanes 4–6) complementary base pairs was reacted with MBP-2CATPase (20 pmol). Lanes 1 and 4, reaction mixture without 2CATPase addition; lanes 2 and 5, boiled reaction mixture without 2CATPase. Asterisks indicate the HEX-labeled strand. (TIF) [file ppat.1005067.s003.tif]

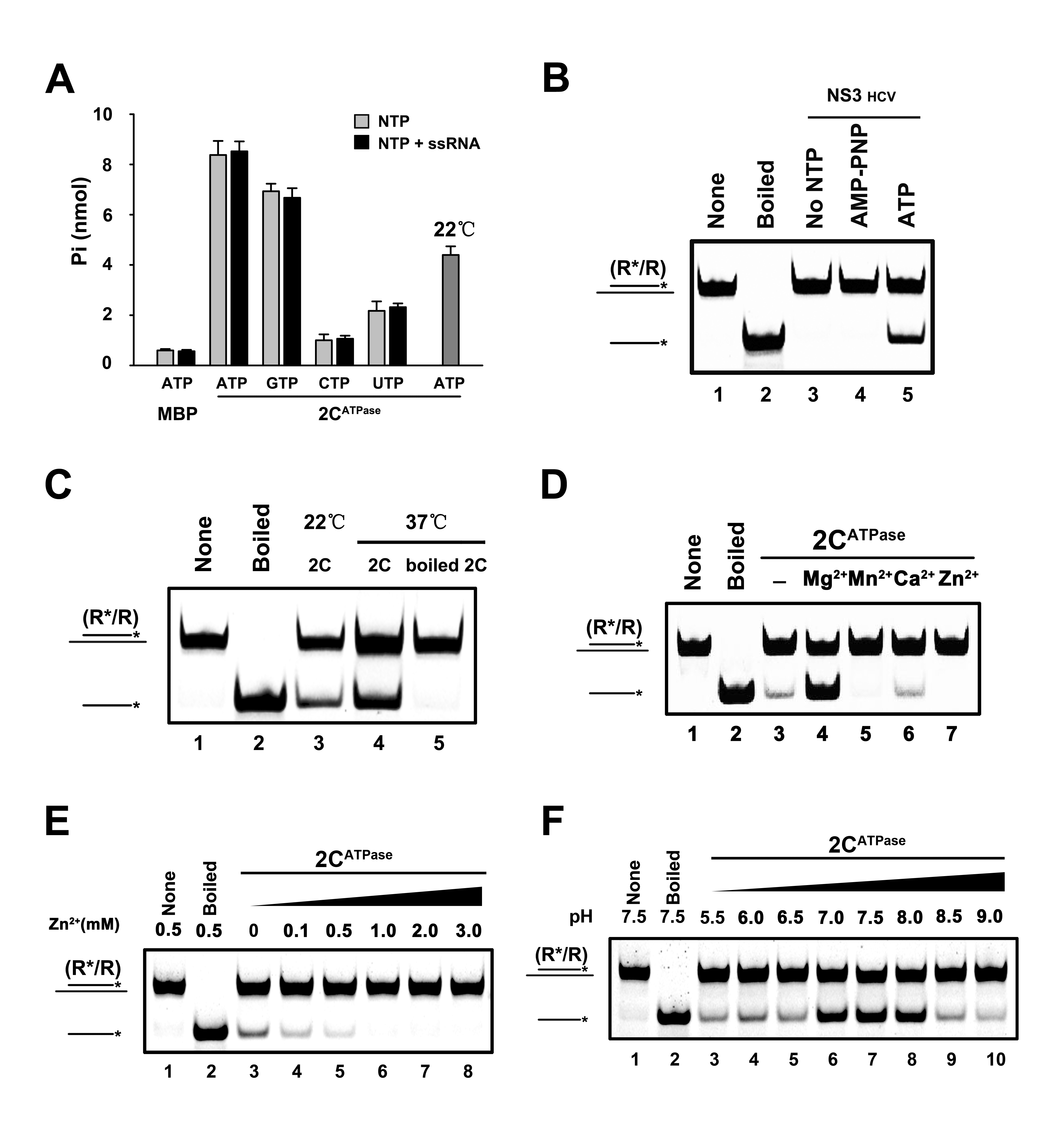

Supplement: S4 Fig — (A) The NTPase activity of MBP-2CATPase on each NTP in the absence or presence of 100 pmol ssRNA was measured at 37°C as nanomoles of released inorganic phosphate as indicated. In addition, the ATPase activity of MBP-2CATPase was measured at 22°C as indicated. MBP alone was used as the negative control. Error bars represent SD values from three separate experiments. (B) The unwinding assays of HCV NS3 using the standard helix substrate were performed in the absence or presence of 5 mM ATP or AMP-PNP as indicated. (C) The unwinding assays of MBP-2CATPase using the standard helix substrate were performed at 22°C or 37°C as indicated. Boiled MBP-2CATPase or no protein supplementation was used as negative control. (D) The standard RNA helix (0.1pmol) was reacted with MBP-2CATPase (20 pmol) in the presence of indicated divalent metallic ions (2.5 mM). (E) The standard RNA helix was reacted with 2CATPase (20 pmol) in varying concentrations of Zn2+. (F) The standard RNA helix was reacted with 2CATPase (20 pmol) at the indicated pH. For (B-F), asterisks indicate the HEX-labeled strand. (TIF) [file ppat.1005067.s004.tif]

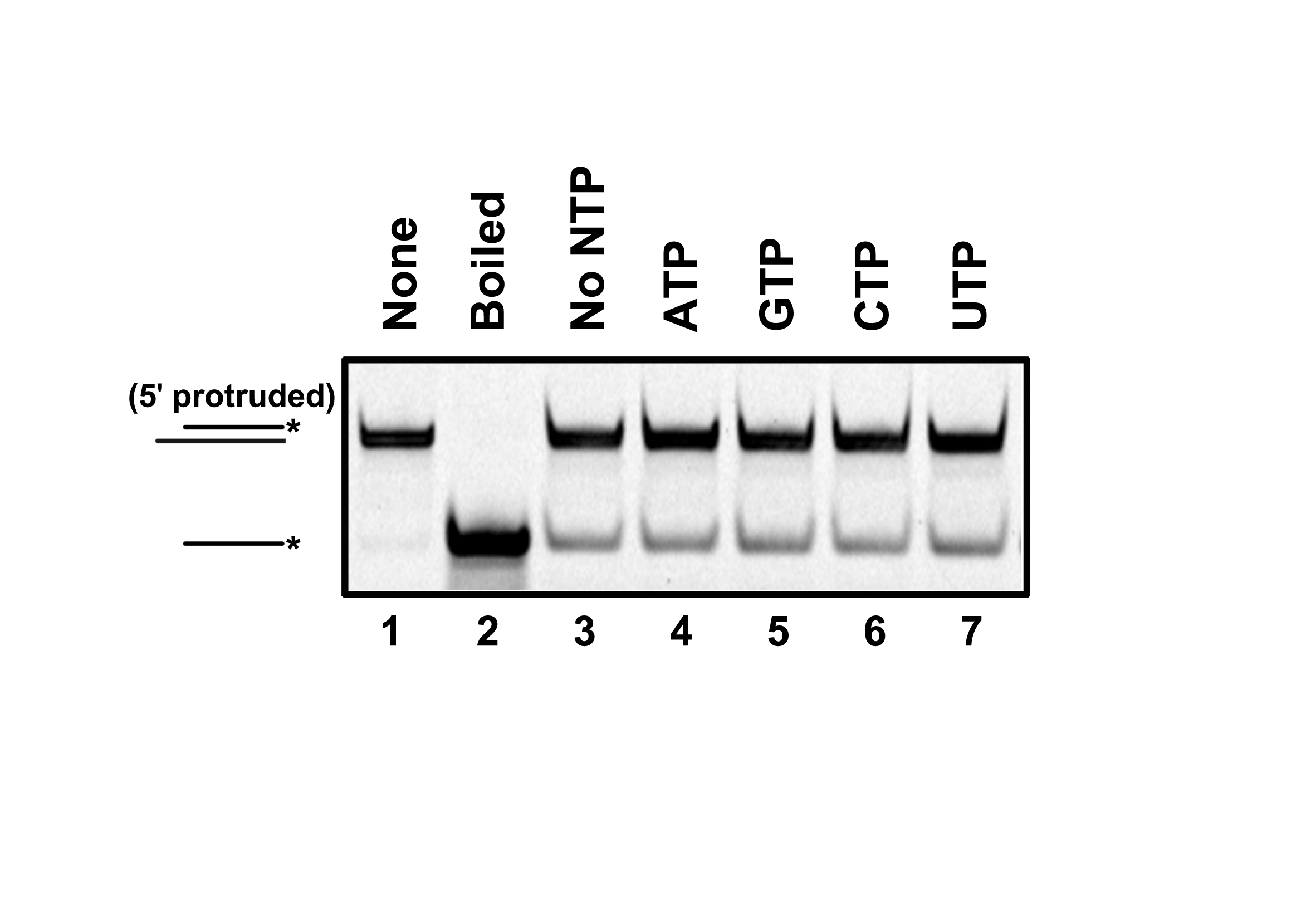

Supplement: S5 Fig — The standard RNA helix was reacted with 2CATPase (20 pmol) in the absence (lane 3) or presence of 5 mM indicated NTP (lanes 4–7). Asterisks indicate the HEX-labeled strand. (TIF) [file ppat.1005067.s005.tif]

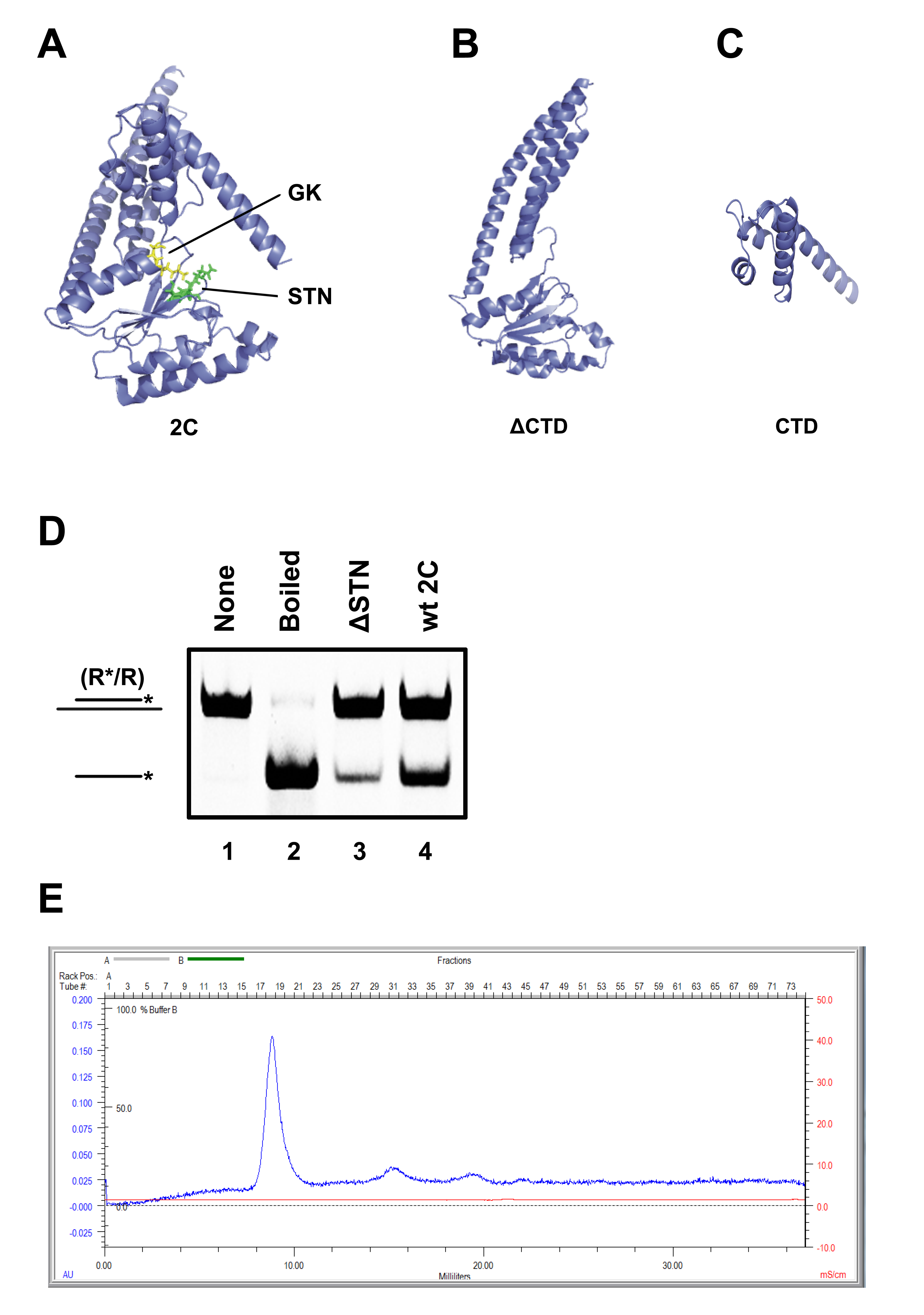

Supplement: S6 Fig — The sites of “GK” in helicase/ATPase motif A and “STN” in helicase motif C are indicated as yellow and green, respectively. (D) The standard RNA helix (0.1 pmol) was reacted with MBP-2CATPase wt (lane 4) or ΔSTN mutant (lane 3) in the presence of 5 mM ATP. Asterisks indicate the HEX-labeled strand. (E) Elution profile of purified MBP-2CATPaseΔCTD from a Superdex 200 increase 10/300 GL column. Protein elution was followed by UV detection at 280 nm. X axis represents the elution volume (in ml). (TIF) [file ppat.1005067.s006.tif]

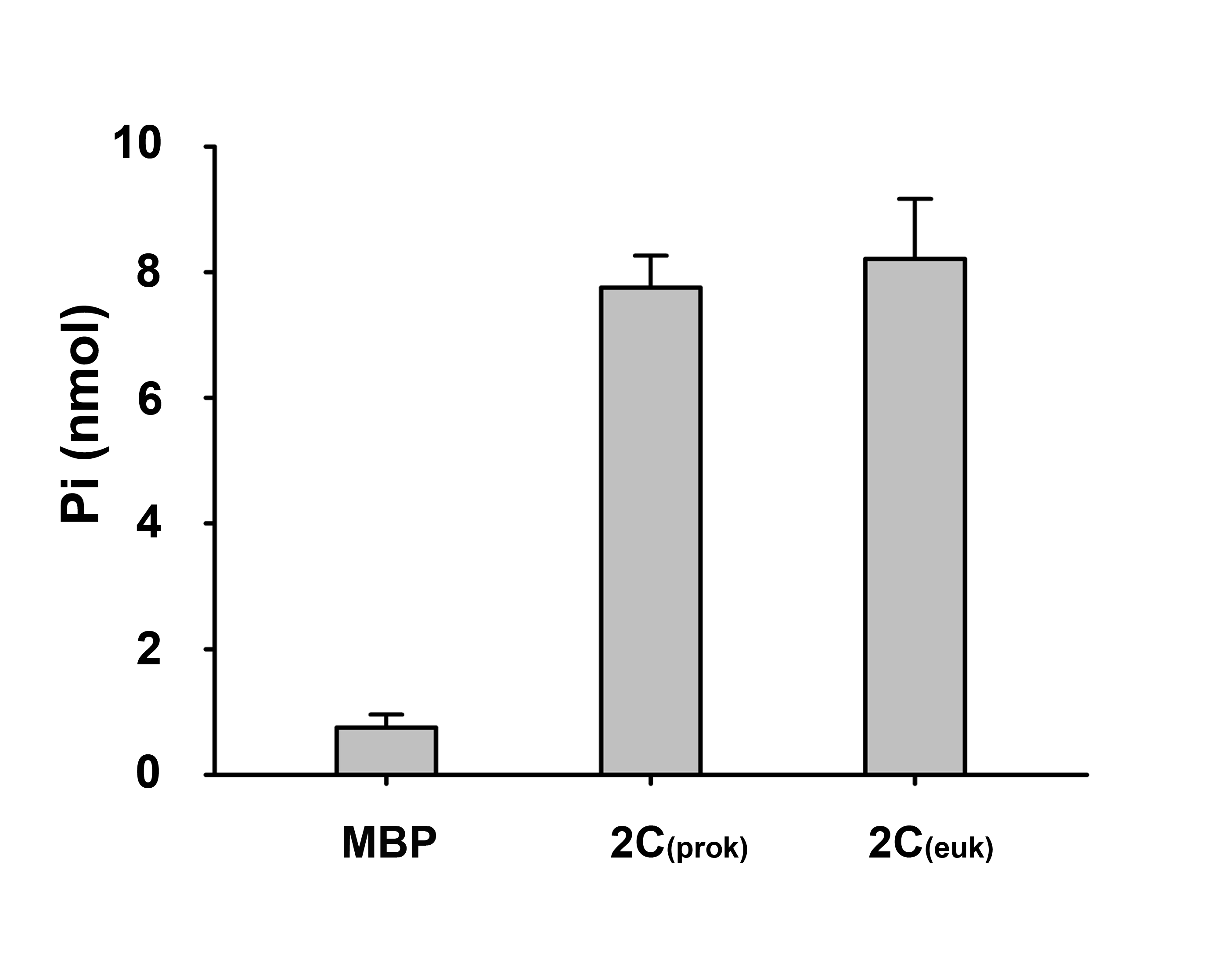

Supplement: S7 Fig — The ATPase activity of indicated proteins was measured at 37°C as nanomoles of released inorganic phosphate. MBP alone was used as the negative control. Error bars represent SD values from three separate experiments. (TIF) [file ppat.1005067.s007.tif]

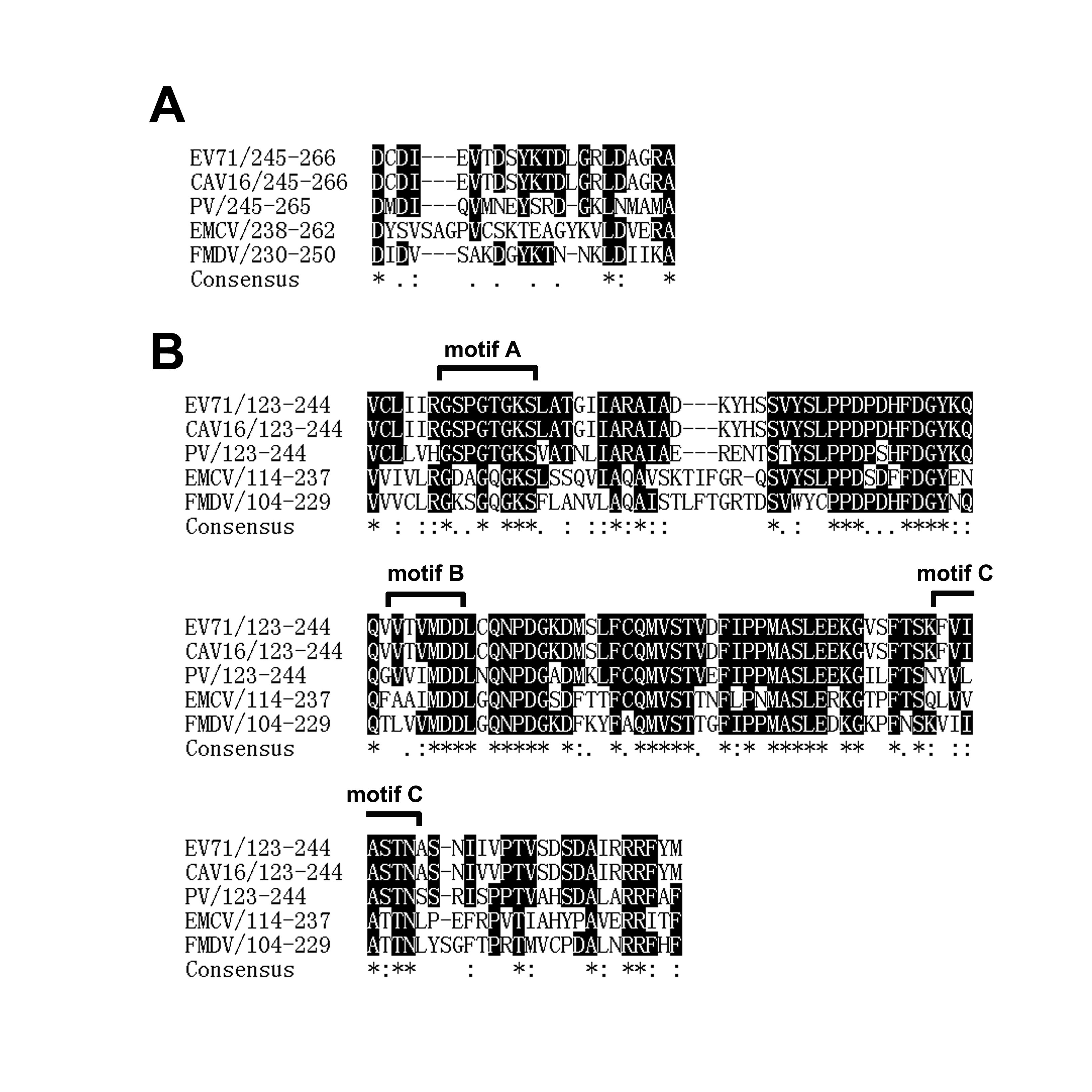

Supplement: S8 Fig — (A) The sequence alignment of the linker region between the HC domain and CTD of 2CATPase proteins from indicated picornaviruses. (B) The sequence alignment of the HC domains of 2CATPase proteins from indicated picornaviruses. EV71, enterovirus 71, genus Enterovirus; CAV16, coxsackie A virus 16, genus Enterovirus; PV, poliovirus, genus Enterovirus; EMCV, encephalomyocarditis virus, genus Cardiovirus; FMDV, foot-and-mouth disease virus, genus Aphthovirus. The alignments were conducted using ClustalX2. The conserved motifs A, B, and C for SF3 helicases are indicated. (TIF) [file ppat.1005067.s008.tif]
