# Supplementary material for: Human Enterovirus Nonstructural Protein 2CATPase Functions as Both an RNA Helicase and ATP-Independent RNA Chaperone
Source: PLoS Pathog. 2015 Jul 28;11(7):e1005067. doi: 10.1371/journal.ppat.1005067 (PMC4517893; doi:10.1371/journal.ppat.1005067)
Supplement: S2 Table — (DOC) [file ppat.1005067.s010.doc]

**Table. S1** **List of Oligonucleotides**

| **Name** | **Sequence (5**′ **to 3**′**)^a^** |
| --- | --- |
| RNA1 | ***CAUUAUCGGAUAGUGGAACCUAGCUUCGACUAUCGGAUAAUC** |
| RNA2 | AAUAAAGAUUAUCCGAUAGUCGAAGCUAGGUUCCACUAUCCGAUAAUGAAAUAA |
| RNA3 | UGUAGUGCUGCCAUGGUGUGGUGGUGGUGGUUGUGGUGGAGCUACGAAC |
| RNA4 | GAUUAUCCGAUAGUCGAAGCUAGGUUCCACUAUCCGAUAAUGAAAUAA |
| RNA5 | AAUAAAGAUUAUCCGAUAGUCGAAGCUAGGUUCCACUAUCCGAUAAUG |
| RNA6 | GAUUAUCCGAUAGUCGAAGCUAGGUUCCACUAUCCGAUAAUG |
| RNA7 | ***GUAACUAUAUUCGUCAUUAUCUCAUUUCCCAAAUUAAUACCAUAAAUUC** |
| RNA8 | UUCGGAUACGGAAUUUAUGGUAUUAAUUUGGGAAAUGAGAUAAUGACGAAUAUAGUUACCGUAUCCGAA |
| DNA1 | AATAAAGATTATCCGATAGTCGAAGCTAGGTTCCACTATCCGATAATGAAATAA |
| DNA2 | ***CACCACAACCACCACCACCACACCATGG** |
| DNA3 | TGTAGTGCTGCCATGGTGTGGTGGTGGTGGTTGTGGTGGAGCTACGAAC |
| Ribozyme | UCUAGACCCACACUCUCUGAUGAGCUCCGUGAGGAGCGAAACUACUUCCUGCA |
| Ribozyme substrate | ***UGUAGUUAAAGUAGUAAGAGUGUCUGCA** |

^a^ * indicate HEX labeled site
