# Supplementary material for: Human Enterovirus Nonstructural Protein 2CATPase Functions as Both an RNA Helicase and ATP-Independent RNA Chaperone
Source: PLoS Pathog. 2015 Jul 28;11(7):e1005067. doi: 10.1371/journal.ppat.1005067 (PMC4517893; doi:10.1371/journal.ppat.1005067)
Supplement: S3 Table — (DOC) [file ppat.1005067.s011.doc]

**Table. S2** List of Primers

| **Primers** | **Sequence (5**′ **to 3**′**)^a^** |
| --- | --- |
| EV71-2C-F | GTCTAGAAGCGCTTCCTGGCTCAAG |
| EV71-2C-R | GAAGCTTTTATTGGAAAAGAGCCTCG |
| GK134AA-F | CTCACCAGGCACTGCTGCGTCTCTAGCCACTGG |
| GK134AA-R | GTGGCTAGAGACGCAGCAGTGCCTGGTGAGCCCC |
| ΔSTN-F | CTAAATTTGTCATCGCAGCCAGTAATATCATAGTGCCAACAG |
| ΔSTN-R | GGCACTATGATATTACTGGCTGCGATGACAAATTTAGAGGTG |
| EoV-2C-F | GGAATTCACTTTCGCCAAAGGTGAAGAC |
| EoV-2C-R | GGTCGACTTAACCCGTGGGTGC |
| ΔCTD-R | GAAGCTTTTACACTTCAATGTCACAGTCC |
| CTD-F | CTCTAGAACAGACTCGTACAAAACAGATC |
| CAV16-2C-F | GGAATTCAGCGCTTCGTGGCTAAAG |
| CAV16-2C-R | CGTCGACTTACTGGAAGAGGGCTTCTATGG |
| HCV-NS3-F | CGAATTCCACGTCGTTGGGCTCTTCCG |
| HCV-NS3-R | CAAGCTTTCAGGTCATGACCTCAAGGTC |
| RNA Template-R | GTAATACGACTCACTATAGGACCGCCACGGTCGCCCG |
| RNA Template-F | TTAAAACAGCCTGTGGGTTGC |
| RNA Primer-F | CTAATACGACTCACTATAGGTTAAAACAGCCTGTGGG |
| RNA Primer-R | TGCCCGCTATTGATCGTGG |

^a^ Underlined characters indicate restriction endonuclease sites
