# Supplementary material for: Human Enterovirus Nonstructural Protein 2CATPase Functions as Both an RNA Helicase and ATP-Independent RNA Chaperone
Source: PLoS Pathog. 2015 Jul 28;11(7):e1005067. doi: 10.1371/journal.ppat.1005067 (PMC4517893; doi:10.1371/journal.ppat.1005067)
Supplement: S1 Text — (DOCX) [file ppat.1005067.s012.docx]

**Supplementary Materials and Methods**

**Silver staining**

Proteins were separated on 10% SDS-PAGE or 5% native-PAGE and visualized by Coomassie blue or the silver staining method. In brief, for the silver staining, gels were first fixed by 50% methanol and 5% v/v acetic acid for 20 min, and 50% methanol for 10 min, followed by sensitization with 0.02% sodium thiosulfate. After that, gels were submerged in silver staining solution (0.1% silver nitrate and 0.08% v/v formaldehyde) for 20 min, followed by rinsing with water for 1 min. Gels were developed by incubating with developer (2% sodium carbonate and 0.04% v/v formaldehyde), and replaced the fresh developer within 30 sec as the developer turning yellow. When protein bands were visible, the gels were transferred immediately to the stop solution containing 5% v/v acetic acid for 10 min, and washed in water for another 5 min. Gels were then permanently stored in 8% v/v glycerol.

**Liquid chromatography-tandem mass spectrometry (LC-MS/MS) analysis**

Puriﬁed MBP-2C^ATPase^ protein sample was subjected to 10% SDS-PAGE. After that, gel band was cut, in-gel digested, and analyzed by LC-MS/MS on 5600 (ABSciex). Data were searched against Uniprot database using Mascot (Matrix Science). The protein knowledgebase (UniProtKB) of *Spodoptera frugiperda* (Taxon identifier 7108) was downloaded from Uniprot, and the amino acid sequence of EV71 MBP-2C^ATPase^ were combined. Trypin was selected as digestion enzyme, and two missed cleavages were allowed. MS mass tolerance was set to 50 ppm, while MS/MS tolerance was set to 0.5 Da. Carbamidomethyl (C) was chosen as fixed modification, while dynamic modifications included were acetylation (protein N-term) and Oxidation (M). Decoy database searching was also performed and the false discovery rates (FDRs) of the peptide-spectra matches was 0.97%. The identified proteins were quantified using the Mascot function Exponentially Modified Protein Abundance Index (emPAI) [[1](#_ENREF_1)].

**Size Exclusion Chromatography Analysis**

The affinity-purified protein sample was applied to a Superdex 200 increase 10/300 GL column (GE Healthcare) pre-equilibrated in buffer containing 50 mM HEPES-KOH (pH 7.5). The buffer and sample were pumped through the column using a [chromatography](http://www.bio-rad.com/en-us/category/products/chromatography) system (BioLogic DuoFlow 10) coupled in line to a UV detector (5 mm flow cell and 254/280nm filter) at a flow rate of 0.5 ml/min. Peak analysis was performed using the ASTRA software package (BioLogic Chromatography Systems).

**Supplementary References**

1. Ishihama Y, Oda Y, Tabata T, Sato T, Nagasu T, Rappsilber J, et al. Exponentially modified protein abundance index (emPAI) for estimation of absolute protein amount in proteomics by the number of sequenced peptides per protein. Mol Cell Proteomics. 2005;4(9):1265-72. doi: 10.1074/mcp.M500061-MCP200. PubMed PMID: 15958392.
